# A comprehensive battery of flow cytometric immunoassays for the *in vitro* testing of chemical effects in human blood cells

**DOI:** 10.3389/fimmu.2023.1327960

**Published:** 2024-01-02

**Authors:** Arkadiusz Pierzchalski, Ana C. Zenclussen, Gunda Herberth

**Affiliations:** ^1^Helmholtz Centre for Environmental Research - UFZ, Department of Environmental Immunology, Leipzig, Germany; ^2^Perinatal Immunology Research Group, Medical Faculty, Saxonian Incubator for Clinical Translation (SIKT), University of Leipzig, Leipzig, Germany

**Keywords:** immune assays, human blood, flow cytometry, chemical testing, activation marker, immune cells, immunomodulation, immunotox

## Abstract

**Background:**

There is a growing need for immunological assays to test toxic and modulatory effects of chemicals. The assays should be easy to use, reproducible and superior to cell line-based assays. We have therefore developed a comprehensive portfolio of assays based on primary human blood cells that are suitable for testing chemical effects.

**Methods:**

The flow cytometry-based assays were designed to target a wide range of human peripheral blood mononuclear cells and whole blood, including T cells, NK cells, B cells, basophils and innate-like T cells such as γδT, MAIT and NKT cells. We have selected a set of activation markers for each immune cell, e.g: CD154 (T cells), CD137, CD107a (NK cells), CD63 (basophils), CD69, CD83 (B cells), CD69, IFN-γ (MAIT cells) and we selected cell specific stimuli: aCD3 antibodies (T cells); E. coli and cytokines IL-12/15/18 (MAIT cells); CpG ODN2006, R848 or aCD40 antibodies (B cells), fMLP or aFcϵR1 (basophils) or K562 cells (NK cells).

**Results:**

By selecting immune cell-specific markers and cell-specific stimuli, we were able to induce particular immune responses from the targeted immune cells. For example, the response to stimulation with anti-CD3 antibodies was in 36.8% of CD107a+CD8+ cells. Cytokine stimulation induced the production of IFN-γ in 30% of MAIT cells. After stimulation with *E. coli*, around 50% of MAIT cells produced TNF. About 40% of basophils responded to aFcƐR1 stimulation. Similar activation ranges were achieved in K562-stimulated NK cells.

**Conclusion:**

Our test portfolio covers the most relevant immune cells present in human blood, providing a solid basis for *in vitro* toxicity and immunomodulatory testing of chemicals. By using human blood, the natural composition of cells found in the blood can be determined and the effects of chemicals can be detected at the cellular level.

## Graphical Abstract

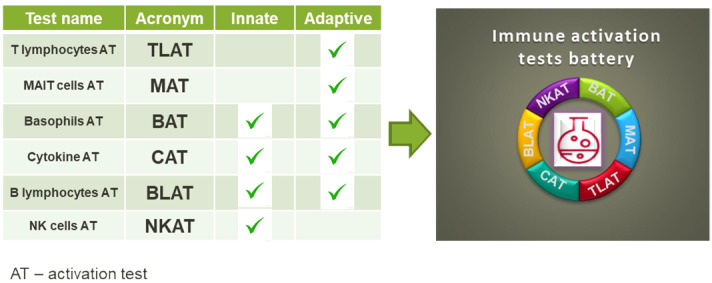

## Introduction

The classical assessment of the public health risk associated with a certain compound and new chemicals placed on the market includes among others acute toxicity, skin irritation, carcinogenicity, reproductive toxicity and toxicity to a specific organ. By now, immunotoxcicity, immunosupression or immunomodulation is not included in the safty test systems of manufacturers. However, exposure to chemicals can target immune cells and lead to health impairment likewise allergies, autoimmune diseases and even cancer (1–3). But, assessing the effects of chemicals on the human immune system is challenging. Assays to investigate the impact of a stressor at immune cell level should cover many different cell types from both, the innate and the adaptive immune system that respond to different stimuli, similar to the response to bacteria or viruses. Recently, immune assays that can validate the influence of specific chemicals on the activity of immune cells (i.e. modulatory effects) were reported (4). These assays include *in vitro* testing of chemicals on keratinocytes (5), dendritic cell lines (6) and lymphocyte cell lines (7). Further, assays for the analysis of immune relevant effects in peripheral blood mononuclear cells (PBMCs) (8) or in the whole blood (9) were developed. For established cell lines, gene activation in reporter assays is employed. However, very often the effects imply activation of a particular pathway in generic cells, and this gives little or no information about its significance in living individuals. The analysis of immune effects in PBMCs focuses on general cell stimulation, such as PMA/ionomycin or LPS, by measuring cytokine secretion. The assays used today for chemical testing either lack the complexity of the cell composition in biological samples that is required to mount a proper immune response (as in the case of cell lines) or are too general in their induction of a cell response e.g. by a generalized readout such as cytokine secretion. To address the need for single cell analysis in the diversity of human peripheral blood cell types, flow cytometry is our method of choice. This technology allows simultaneous analysis of the majority of immune cell populations based on their precise determination of phenotype as well as response to stimuli, which is possible by careful selection of lineage and activation markers (10). The use of flow cytometry for single cell analysis allows high-throughput screening of chemicals (11), which promises to identify affected cells and determine which signaling pathway may have been affected (12). Here, we propose a battery of *in vitro* assays to address the diversity of immune responses in human peripheral blood. Our test portfolio covers the activation of T cells and their subtypes including T helper (Th) cell subtypes, γδT cells, MAIT and NKT cells. In addition, we have established assays for the activation of NK and B cells as well as basophils (**Graphical Abstract**). In addition to basophil testing, for which whole blood is used, we established tests for isolated human peripheral blood mononuclear cells (PBMC). The advantage of the established tests is that they are fast, sensitive and easy to perform.

**Figure 1:**
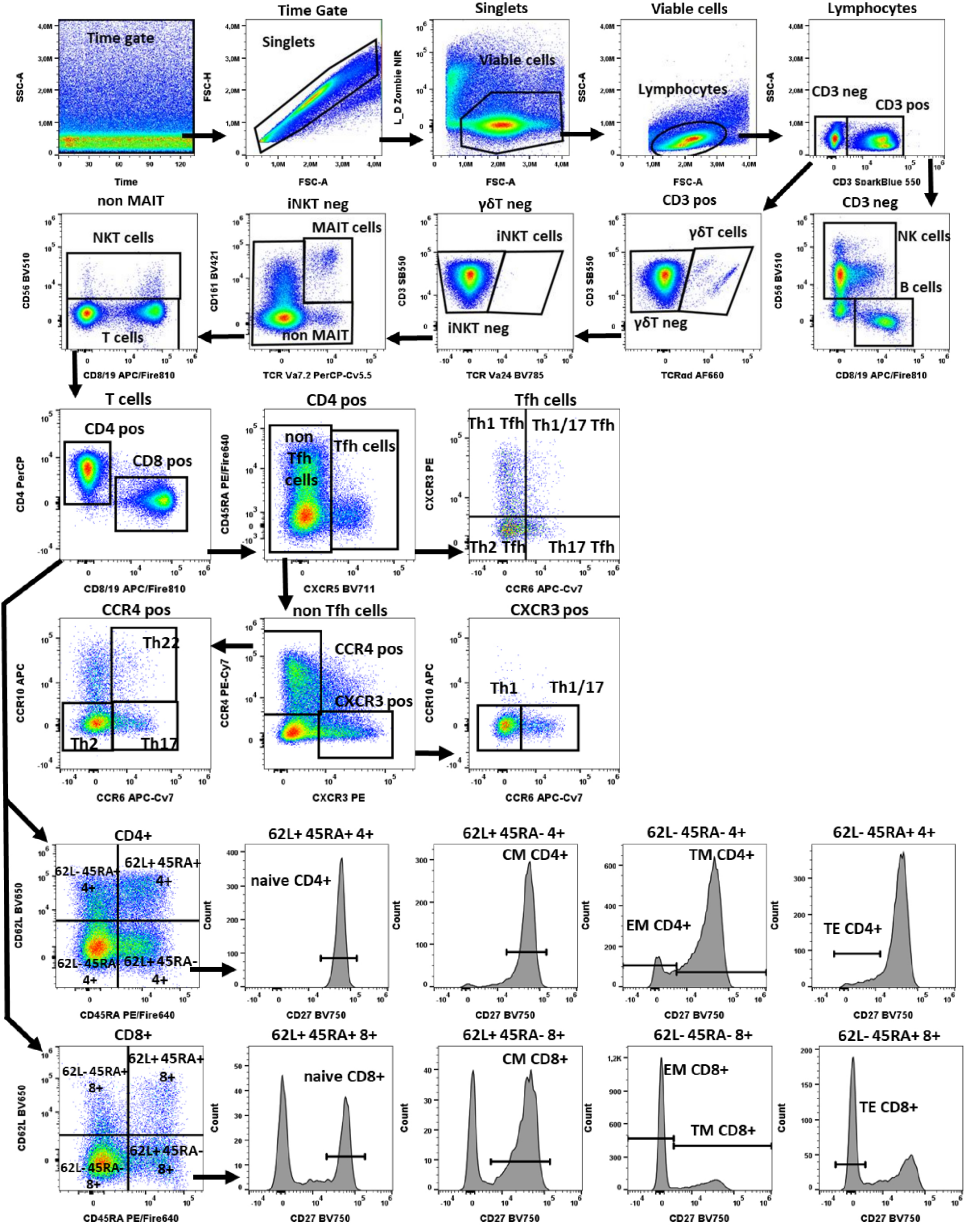
Gating strategy in TLAT. We used a time gate to focus on events from stable flow stream. After doublet exclusion, viable cells were selected by using NIR Zombie for dead cell exclusion. Lymphocytes were gated based on FSC-A vs SSC-A. In CD3 neg lymphocytes, B cells were identified by CD19 and NK cells by CD56 expression. In CD3 pos cells γδTCR expressing cells were defined as γδT cells. Furthermore, in the γδT neg population iNKT cells were identified by expression of TCR Va24. Next, in the iNKTneg cells, MAIT cells were selected as double positive for CD161 and TCRVa7.2 expression. In the remaining cells, NKT cells were selected as CD56 expressing cells. Within T cells, conventional T helper (Th) and cytotoxic T (Tc) cells, were identified by the expression of CD4 and CD8 respectively. In both populations, CD45RA, CD62L and CD27 were used to describe: naive T cells (CD45RA+CD62L+CD27+), central memory (CM) (CD45RA-CD62L+CD27+), translational memory (TM) (CD45RA-CD62L-CD27+), effector memory (EM) (CD45RA-CD62L-CD27-), and terminal effector (TE) (CD45RA+CD62L-CD27-) cells. In addition, CD4 pos were subdivided into Tfh (T follicular helper cells) expressing CXCR5+ and non-Tfh cells (CXCR5- cells). Tfh cells were classified according to the expression of CXCR3 and CCR6 into: Th1 Tfh cells (CXCR3+ CCR6-); Th1/17 Tfh (CXCR3+CCR6+); Th17 Tfh (CXCR3-CCR6+) and Th2 Tfh (CXCR3-CCR6-). Non-Tfh cells cells, were further classified by the expression of CXCR3 and CCR4 as: Th1 (CXCR3+CCR6-); Th1/17 (CXCR3+CCR6+); Th2 (CCR4+CCR6-CCR10-), Th17 (CCR4+CCR6+CCR10-) and Th22 (CCR4+CCR6+CCR10+) cells. A representative color dot plot is shown (n=4). The expression of activation markers on main lymphocyte populations, Th subtypes and T cells maturation subsets is shown in **Figure 2** by tSNE analysis and **Supplementary Figure 1**.

## Methods

**Supplementary Table 1** presents the summary of all activation tests including stimulus type, targeted cells, activation time needed to exert the effect, number of markers used for the antibody panel as well as activation markers selected for cell-type specific activation.

### PBMC and whole blood collection

Pseudonymous buffy coat samples and heparin-collected whole blood from healthy adult volunteers were obtained from the blood bank at the University of Leipzig, after written informed consent. The study was approved by the Ethics Committees of the University of Leipzig (#079-15-09032015). PBMCs were isolated by density-gradient centrifugation using Ficoll Paque Plus (Cytiva Sweden AB, Uppsala, Sweden). Cells were then stored in 10% DMSO (Sigma-Aldrich, St. Louis, US) in fetal bovine serum (FBS) (Sigma-Aldrich, St. Louis, US) at - 150 °C until use. Before treatment, PBMCs were thawed and cultured in IMDM (Gibco, Thermo Fisher Scientific, Waltham, US) supplemented with 10% fetal bovine serum (Sigma-Aldrich, St. Louis, US), 1X Penicillin-Streptomycin (Gibco, Thermo Fisher Scientific, Waltham, US), and 50 μM β-mercaptoethanol (Sigma-Aldrich, St. Louis, US) (complete IMDM medium). Assays were performed with PBMCs or whole blood from at least 4 different donors, results from one representative donor are shown.

### T Lymphocyte activation test (TLAT)

#### PBMC treatment and staining

PBMCs were plated at 10^6^ cells/well in 100 µl complete IMDM medium in U-bottom 96-well microplates (Greiner Bio-One, Frickenhausen, Germany) for at least 2 h at 37°C in a 5% CO_2_ incubator. PBMCs were stimulated in a total volume of 200 µl complete IMDM medium with anti-CD3 antibodies (0.5 ng/ml, Clone: OKT-3, BioLegend, San Diego, CA, USA) for 6 h to induce activation. For the last 4 h of incubation Brefeldin A (10µg/ml) and monensin (1 µM) with anti-CD107a AlexaFluor 647 antibodies (1:1600) in additional 22,5 µl of complete IMDM medium were used.

**Figure 2:**
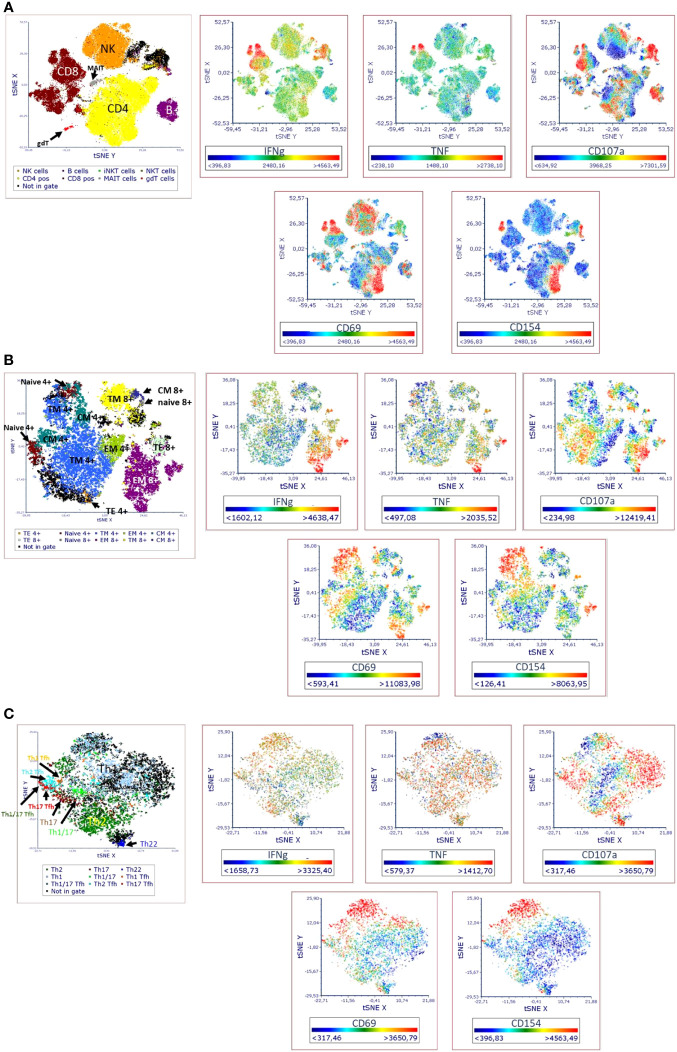
Visual distribution of phenotypic and activation markers (t-SNE analysis) in TLAT in **(A)** lymphocytes **(B)** maturation subsets of Th and Tc cells **(C)** Th cells subsets. t-SNE analysis for a full stained anti-CD3 stimulated PBMC sample was performed in FCS Express 7. Parameters for t-SNE analysis: in **(A)** sample size of 100,000 total events for lymphocytes, the downsampling was set as interval, with iteration number: 500, perplexity: 50, and approximation: 0.5; in **(B)** sample size of 10,000 total events for CD3+ cells, the downsampling was set as weighted density with weight: 0 and alpha: 5 with iteration number: 1000, perplexity: 30, and approximation: 0.5; in **(C)** sample size of 5,000 total events for CD4+ cells, the down-sampling was set as weighted density with weight: 10 and alpha: 5 with iteration number: 1000, perplexity: 50, and approximation: 0.5. Furthermore, in each case the opt-SNE and the estimation for unsampled events were chosen and the Barnes-Hut Approximation was performed. The results were visualized in 2D t-SNE maps. The colors indicate the cell populations which have been identified in t-SNE by backgating. A red color indicates higher expression of a marker and a blue indicates lower/no expression. A representative t-SNE analysis is shown (n=4). Manual gating for each identified cell population and corresponding activation marker is shown in **Supplementary Figure 1**.

Following 6 h stimulation, PBMCs were transferred to V-bottom 96-well microplates (Thermo Fisher Scientific Waltham, US). To discriminate between viable and dead cells, the samples were stained in 100 µl of with fixable viability dye-Zombie NIR™ (BioLegend) in PBS for 15 min at RT, thereafter cells were pre-stained with 40 µl anti-CXCR3, -CXCR5 and -TCRgd antibodies in staining buffer (1% FBS in PBS) for 10 min at RT which was followed up by anti-CD3, CD4, CD8, CD19, CD56, CD161, TCRVα7.2, TCRVα24, CD45RA, CD62L, CD27, CCR4, CCR6, CCR10 antibody surface staining in 60 µl of staining buffer for additional 20 min at RT (**Supplementary Table 2**). After surface staining PBMCs were fixed using FACS™ Lysing Solution (BD Biosciences, San Jose, US) for 10 min, and permeabilized with FACS™ Permeabilizing Solution 2 (BD Biosciences, San Jose, US) for further 10 min. Finally, PBMCs were stained for activation and intracellular markers (CD69, CD154, TNF-α, and IFN-γ) in 100 µl staining buffer for 20 min at RT (**Supplementary Table 2**). A representative gating strategy for TLAT is shown in **Figure 1** (FlowJo^®^ v10 software). To present the expression of all activation markers on all T cell subtypes, tSNE analysis (FCS Express 7, *De Novo* Software) was performed (**Figure 2**).

### MAIT cells activation test (MAT)

#### PBMC treatment and staining

PBMCs were plated at 10^6^ cells/well in 100 µl complete IMDM medium in U-bottom 96-well microplates (Greiner Bio-One, Frickenhausen, Germany) for at least 2 h at 37°C in a 5% CO_2_ incubator. In order to specifically activate MAIT cells within PBMCs, *Escherichia coli* K12 MG1655 samples (*E. coli* K12) were prepared as previously described (13) and stored at – 80°C until use. Prior experiments determined the optimal bacteria concentration, indicating 10 bacteria per cell (10 BpC) as sufficient to activate MAIT cells (14). PBMCs were stimulated with *E. coli* K12 (10 BpC) in a total volume of 200 µl complete IMDM medium for 6 h. Brefeldin A (10 μg/mL) in 22.5 µl of complete IMDM medium was added for the final 4 h of incubation.

**Figure 3:**
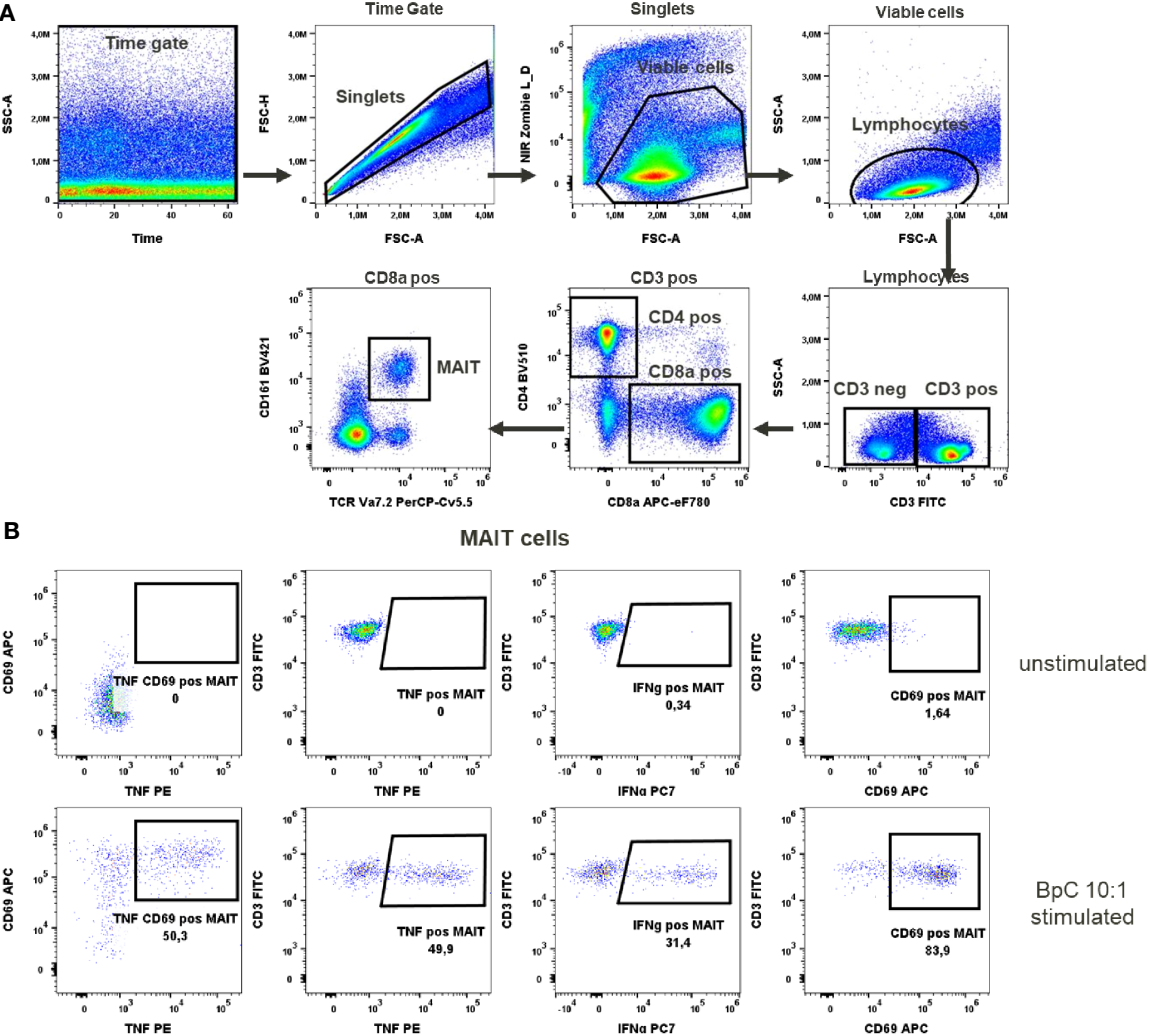
MAIT cells activation test. **(A)** Gating strategy: Time gate was used to focus on events from stable flow stream. After doublet exclusion, viable cells were selected as NIR Zombie negative. Lymphocytes were gated based on FSC-A vs SSC-A. In CD3 positive cells, CD4 and CD8a expressing cells were gated. In CD8a positive cells MAIT cells were identified based on CD161 and TCR Va7.2 expression. **(B)** Expression of activation markers in unstimulated and *E.coli* stimulated (BpC 10:1) MAIT cells. The numbers shown in the dot plots indicate the percentage of positive events for each marker. A representative color dot plot is shown (n=6).

Following 6 h stimulation, PBMCs were transferred to V-bottom 96-well microplates (Thermo Fisher Scientific Waltham, US). To discriminate between viable and dead cells, the samples were stained with 100 µl fixable viability dye-Zombie NIR™ (BioLegend, San Diego, CA, USA) in PBS for 15 min at RT, thereafter stained for surface markers (CD3, CD4, CD8a, CD161, TCRVα7.2) in 100 µl staning buffer for 20 min at RT (**Supplementary Table 3**). After surface staining PBMCs were fixed using FACS™ Lysing Solution (BD Biosciences, San Jose, US) for 10 min, and permeabilized with FACS™ Permeabilizing Solution 2 (BD Biosciences, San Jose, US) for further 10 min. Finally, PBMCs were stained for activation and intracellular markers (CD69, TNF-α, and IFN-γ) in 100 µl staining buffer for 20 min at RT (**Supplementary Table 3**). A representative gating strategy for MAT is shown in **Figure 3** (FlowJo^®^ v10 software).

### Basophils activation test (BAT)

#### Whole blood treatment and cell staining

**Figure 4:**
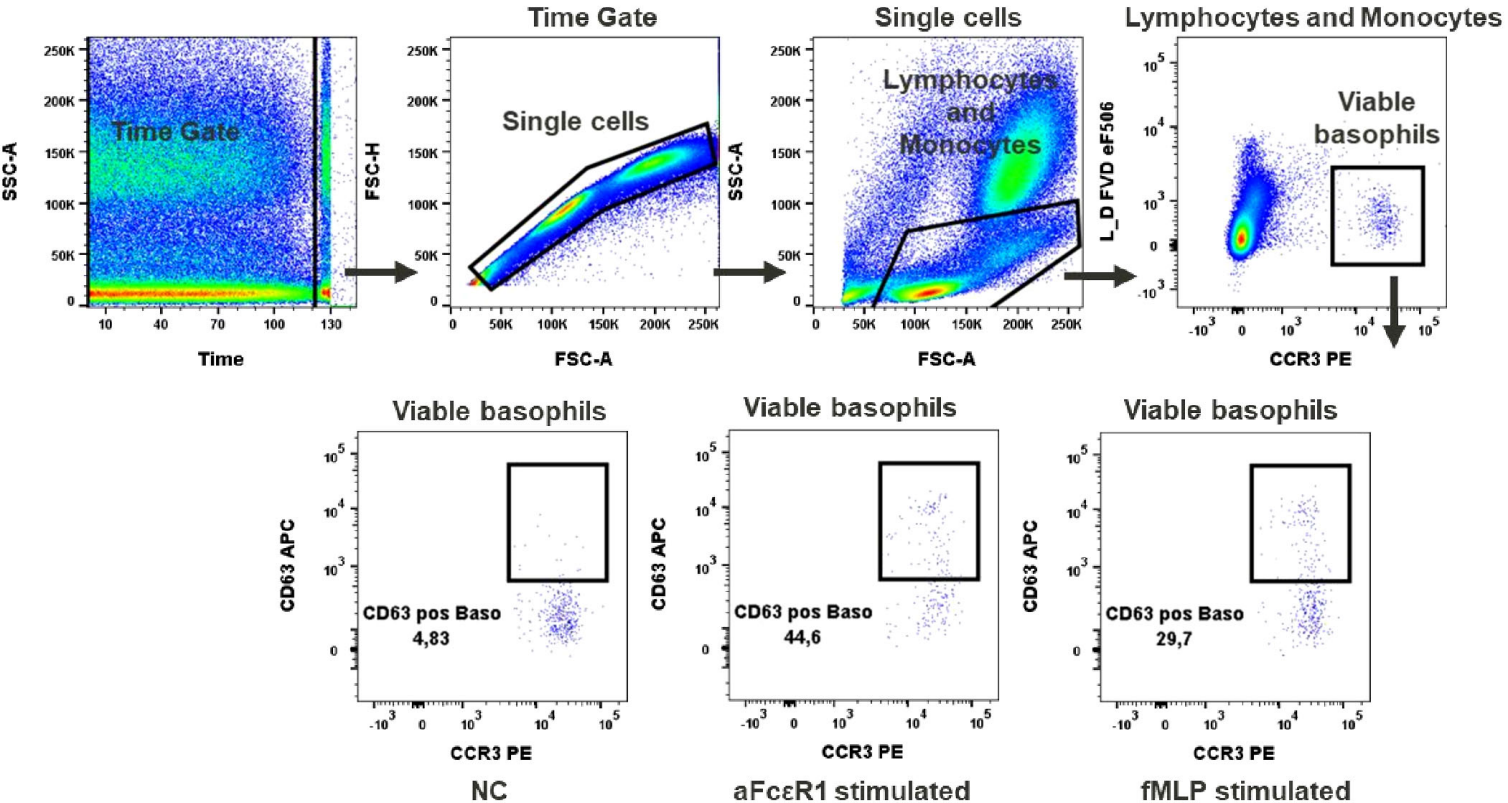
Basophils activation test. Gating strategy: Time gate was used to focus on events from stable flow stream. Doublets were excluded in the FSC-A vs FSC-H plot. Lymphocytes/monocytes were gated in FSC-A vs SSC-A. Viable basophils were identified as FVD eF506 negative and CCR3 positive cells. Percentage of CD63 positive events shows activation of basophils upon stimulation with fMLP or anti-FcϵR1 antibodies, NC (negative control). A representative color dot plot is shown (n=8).

100 μL of blood were transferred in FACS 5 mL polystyrene round-bottom tubes (Falcon, Corning, New York, US). For this assay, a shorter time for activation was selected due to the shorter half-life of basophils compared to lymphocytes. As negative control, blood exposed to BAT buffer only was used (72.5 µg/ml MgCl_2_ (Sigma-Aldrich), 100 µg/ml CaCl_2_ (Sigma-Aldrich), 1ng/ml IL-3 in PBS). Basophils were activated through the addition of anti-FcϵR1α antibody (0.1 μg/mL, BioLegend) or with *N*-Formylmethionylleucyl-phenylalanine (fMLP 0.05 μM, Sigma-Aldrich, St. Louis, US) in 50 µl of BAT buffer for 25 min with simultaneous staining with anti-CCR3 and anti-CD63 antibodies in 50 µl of BAT buffer (**Supplementary Table 4**). After 25 min, the reaction was stopped by adding EDTA 3.8% (Gibco, Thermo Fisher Scientific, Waltham, US). Erythrocytes were then lysed twice using 2 ml erythrocytes lysis buffer (NH_4_Cl – Sigma-Aldrich, NaCO_3_ – KMF Laborchemie, Lohmar, Germany, EDTA – Thermo Fisher Scientific) and incubated 10 min at RT. To discriminate between viable and dead cells, 50 µl of fixable viability dye-eFluor™ 506 (Thermo Fisher Scientific, Waltham, US) in PBS was added and incubated for 20 min at 4°C. Thereafter, cells were fixed in 200 µl paraformaldehyde 1% (Sigma-Aldrich) and analyzed by flow cytometry using FACS Canto™ II (BD Biosciences, San Jose, US). A representative gating strategy for BAT is shown in **Figure 4** (FlowJo^®^ v10 software).

### Cytokines activation test (CAT)

#### PBMC treatment and staining

PBMCs were plated at 10^6^ cells/well in 100 µl complete IMDM medium in U-bottom 96-well microplates (Greiner Bio-One, Frickenhausen, Germany) for at least 2h at 37°C in a 5% CO_2_ incubator. In order to specifically stimulate MAIT, NK, NKT and γδT cells, PBMC were incubated with IL-12/IL-15/IL-18 (10/25/25 ng/ml) in a total volume of 200 µl complete IMDM medium for 20h (Miltenyi/Miltenyi/R&D Systems). Brefeldin A (10 μg/mL) in 22.5 µl of complete IMDM medium was added for the final 4h of incubation.

**Figure 5:**
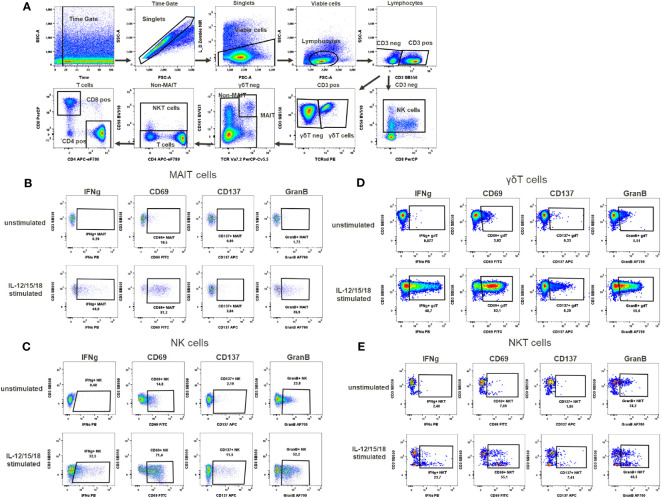
Cytokines activation test. **(A)** Gating strategy: Time gate was used to focus on events from stable flow stream. After doublet exclusion, viable cells were selected as NIR Zombie negative. Lymphocytes were gated based on FSC-A vs SSC-A. In CD3 negative lymphocytes, NK cells were identified by CD56 expression. In CD3 positive cells, γδT cells were defined by the expression of TCRγδ. In the TCRγδ negative population, MAIT cells were identified as double positive for CD161 and TCRVa7.2. In the non-MAIT population, NKT cells were identified by CD56 expression and remaining cells were defined as T cells expressing CD4 or CD8. Expression of activation markers (IFNg, CD69, CD137, GranB) after IL-12/15/18 stimulation or without stimulation on **(B)** MAIT cells **(C)** NK cells **(D)** γδT cells **(E)** NKT cells. The percentage of positive events is shown below each gate. A representative color dot plot is shown (n=4).

Following 20h stimulation, PBMCs were transferred to V-bottom 96-well microplates (Thermo Fisher Scientific Waltham, US). To discriminate between viable and dead cells, the samples were stained in 100 µl fixable viability dye-Zombie NIR™ (BioLegend, San Diego, CA, USA) in PBS for 15 min at RT, thereafter stained for surface markers (CD3, CD4, CD8, CD56, CD161, TCRgd, TCRVα7.2) in 100 µl staining buffer for 20 min at RT (**Supplementary Table 5**). After surface staining PBMCs were fixed using FACS™ Lysing Solution (BD Biosciences, San Jose, US) for 10 min, and permeabilized with FACS™ Permeabilizing Solution 2 (BD Biosciences, San Jose, US) for further 10 min. Finally, PBMCs were stained for activation and intracellular markers (CD69, CD137, Granzyme B and IFN-γ) in 100 µl staining buffer for 20 min at RT (**Supplementary Table 5**). A representative gating strategy for CAT is shown in **Figure 5** (FlowJo^®^ v10 software).

### B lymphocytes activation test (BLAT)

#### PBMC treatment and staining

PBMCs were plated at 10^6^ cells/well in 100 µl of complete IMDM medium in U-bottom 96-well microplates (Greiner Bio-One, Frickenhausen, Germany) for at least 2h at 37°C in a 5% CO_2_ incubator. In order to activate B lymphocytes specifically, PBMCs were stimulated with 0.1 µM CpG ODN2006 (Miltenyi Biotec, Bergisch-Gladbach, German), 0.25 µg/ml goat anti-IgM/IgG serum (BioLegend, San Diego, CA, USA), 0.5 µg/ml R848 (Sigma-Aldrich, St. Louis, US) or 1 µg/ml anti-CD40 antibodies (BioLegend, San Diego, CA, USA) in a total volume of 200 µl complete IMDM medium for 4h.

**Figure 6:**
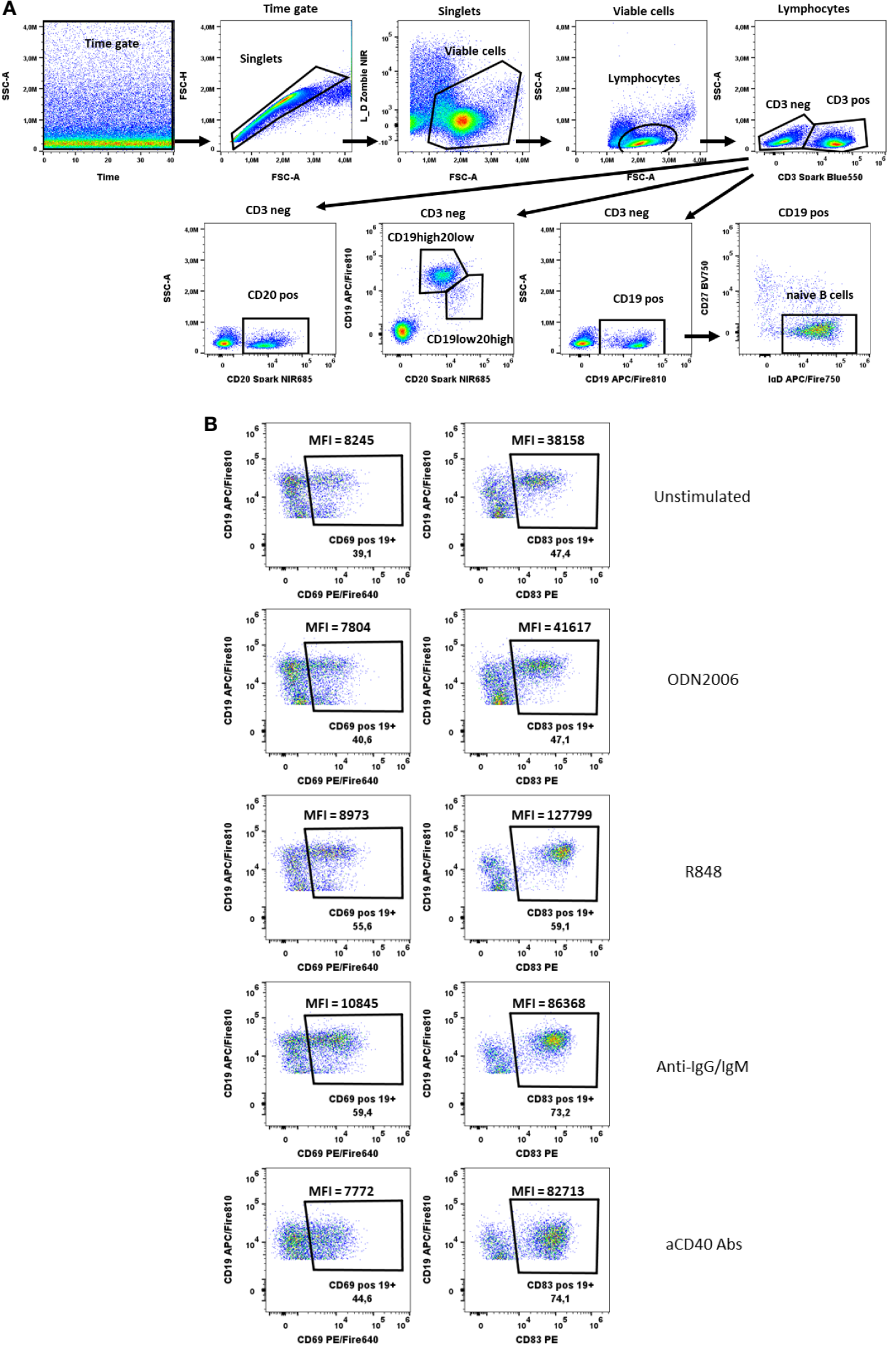
B cells activation test. **(A)** Gating strategy: Time gate was used to focus on events from stable flow stream. After doublets exclusion, viable cells were selected as NIR Zombie negative. Lymphocytes were gated based on FSC-A vs SSC-A. In CD3 negative cells, CD19+ cells, CD20+ cells or CD19+/CD20+ cells were shown. For CD19+ cells, cells negative for CD27 and positive for IgD were defined as naive B cells. **(B)** Expression of activation markers (CD69, CD83) on CD19+ cells after stimulation with ODN2006 (0.1 µM), R848 (0.5 µg/ml), anti-IgG-IgM serum (0.25 µg/ml), anti-CD40 Abs (0.25 µg/ml). MFI values indicate the intensity of activation marker expression. A representative color dot plot is shown (n=4).

Following 4h stimulation, PBMCs were transferred to V-bottom 96- well microplates (Thermo Fisher Scientific Waltham, US). To discriminate between viable and dead cells, the samples were stained with 100 µl fixable viability dye-Zombie NIR™ (BioLegend, San Diego, Ca, USA) for 15 min at RT, thereafter stained for surface markers (CD3, CD19, CD20, CD27, IgD) and activation markers (CD69, CD83) in 100 µl staining buffer for 20 min at RT (**Supplementary Table 6**). A representative gating strategy for BLAT is shown in **Figure 6** (FlowJo^®^ v10 software).

### NK cells activation test (NKAT)

#### PBMC treatment and staining

PBMCs were plated at 250K cells/well in 100 µl complete IMDM medium in U-bottom 96-well microplates (Greiner Bio-One, Frickenhausen, Germany) overnight at 37°C in a 5% CO_2_ incubator. To provide NK-specific stimulus K562 cells (human erythroleukemia cells) were used. K562 cells were cultured in RPMI1640 medium supplemented with 10% FBS 1X Penicillin-Streptomycin (Gibco, Thermo Fisher Scientific, Waltham, US), and 50 μM β-mercaptoethanol. PBMCs were co-exposed to K562 cells at ratios effector (NK) – target (K562): 25:1; 12.5: 1; 6.25:1 in a total 200 µl of complete IMDM medium for 6h. Brefeldin A (10 µg/ml), monensin (1 µM) and anti-CD107a antibodies were added in 22.5 µl complete IMDM medium for the final 4h incubation.

**Figure 7:**
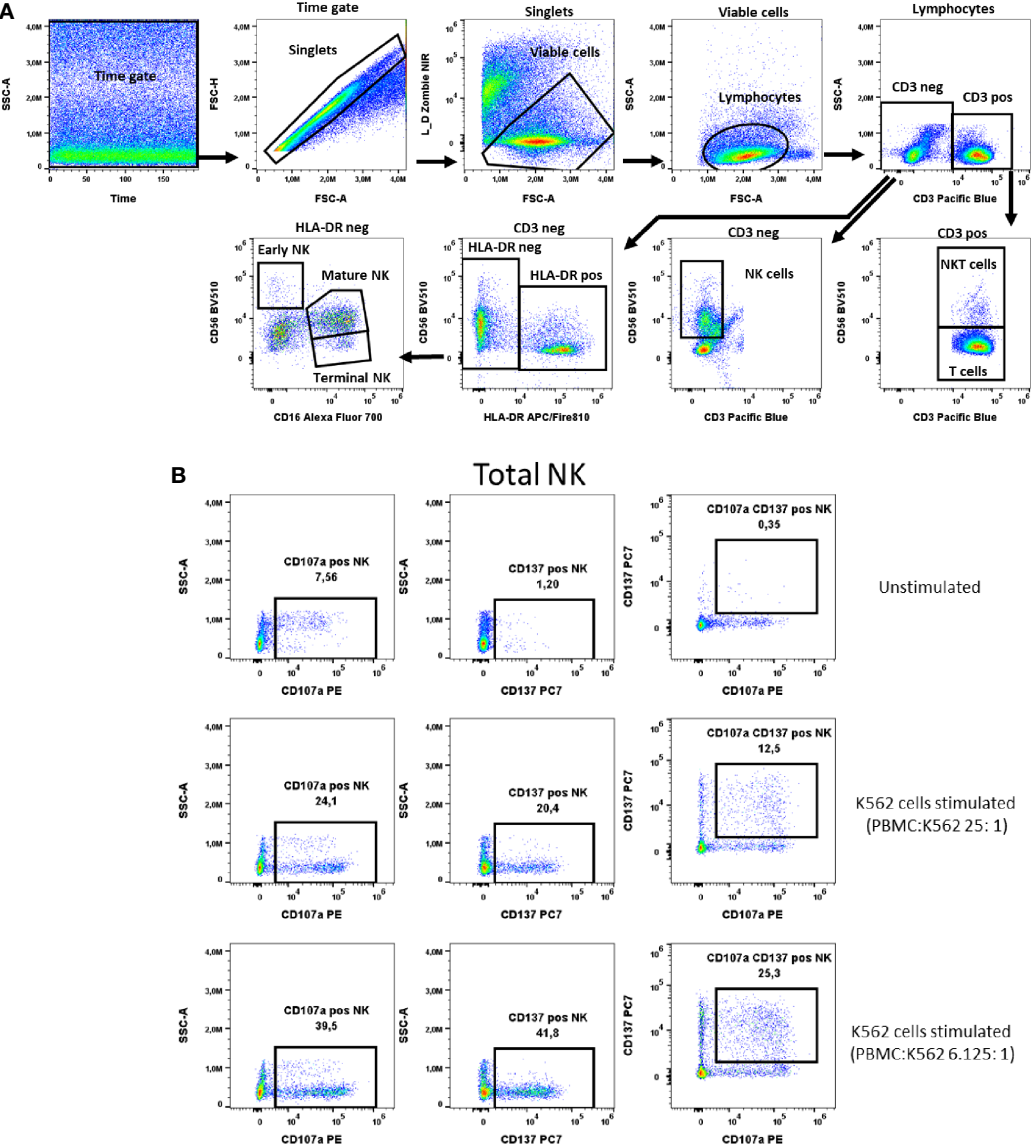
NK cells activation test. **(A)** Gating strategy: Time gate was used to focus on events from stable flow stream. After doublet exclusion, live cells were selected as NIR Zombie negative. Lymphocytes were gated based on FSC-A vs. SSC-A. In the CD3 negative population, CD56 expressing cells were defined as NK cells (total NK). In the CD3 positive population, CD56 expressing cells were defined as NKT cells. In CD3neg cells, HLA-DRneg cell population was identified and by expression of CD16 vs CD56 three NK subpopulations were identified: early (CD56highCD16low), mature (CD56lowCD16high) and terminal (CD56negCD16high) NK. **(B)** Expression of activation markers on total NK cells. PBMCs were stimulated with K562 cells at different ratios. The numbers in the dot plots indicate the percentage of positive events. In plots showing CD137 vs. CD107a expression, only numbers relating to the double positive population are shown. A representative colored dot plot is shown (n=4). The expression of CD137 and CD107a on NK cell subtypes is shown in **Supplementary Figure 4**.

Following 6h stimulation, PBMCs were transferred to V-bottom 96-well microplates (Thermo Fisher Scientific Waltham, US). To discriminate between viable and dead cells, the samples were stained with fixable viability dye-Zombie NIR™ (BioLegend, San Diego, CA, USA) in PBS for 15 min at RT, thereafter stained for surface markers (CD3, CD16, CD56, HLA-DR) in 100 µl staining buffer for 20 min at RT (**Supplementary Table 7**). After surface staining PBMCs were fixed using FACS™ Lysing Solution (BD Biosciences, San Jose, US) for 10 min, and permeabilized with FACS™ Permeabilizing Solution 2 (BD Biosciences, San Jose, US) for further 10 min. Finally, PBMCs were stained for activation marker (CD137) in 100 µl staining buffer for 20 min at RT (**Supplementary Table 7**). A representative gating strategy for NKAT is shown in **Figure 7** (FlowJo^®^ v10 software).

### Proof of concept with immunomodulatory substances

To simulate immunomodulation in our established assays, we performed the TLAT, MAT, CAT, BLAT and NKAT assays with known immunomodulatory substances, i.e. lipopolysaccharide (LPS), rapamycin (RAPA) and cyclosporin A (CsA). Stimulation in the assays was performed as described above. LPS (100 ng/ml), RAPA (50 nM) and CsA (100 ng/ml or 1 µg/ml) were added at the same time as the corresponding stimulus in the assay and the cells were then incubated for 4h (BLAT) 6h (TLAT, MAT, NKAT) or 20h (CAT), followed by the procedure described above.

### Flow cytometric analysis

Excepting BAT assay, which was analyzed on FACS Canto™ II (BD Biosciences, San Jose, US), for all assays flow cytometrical acquisition was performed using the spectral flow cytometer Cytek Aurora (Cytek Biosciences, California, US). The instrument is equipped with 3 lasers (405, 488 and 635 nm) and 38 detectors. Daily QC was performed using SpectroFlo^®^ QC beads prior to acquiring samples to ensure that the cytometer was performing optimally. Daily QC assessed the instrument’s optical alignment and the system performance drift by measuring %rCVs and gains needed to place the beads at the target locations established for each detector. During the typical QC protocol, Laser delays and area scaling factors were also optimized, and gain settings adjusted to account for day-to-day instrument variability. We used default instrument settings for PBMCs saved in Cytek Assay Setting (CAS) on all Cytek Aurora systems which are suggested by Cytek as the most accurate for proper fluorochrome recognition. The use of daily QC beads control and Cytek Assay Settings ascertains high reproducibility of results for inter-experiments comparison as well as for inter-laboratory data reproducibility for Cytek Aurora Instruments. The instrument configuration and the selected fluorochromes with their peak emission are shown in **Supplementary Table 8**. Additional information concerning instrument setup including spectral unmixing as well as N-by-N bivariate plots are provided in **Supplementary Figures S11-S16**. A minimum of 100,000 viable T cells (according to Zombie NIR™ staining) was acquired per sample.

## Results

**Supplementary Table 9** in the **Supplementary Material** shows the individual results and mean ± SD of the analyzed donors in each assay. Data of the main immune cell populations are presented after stimulation with the corresponding stimulus giving insights into the variability of immune responses of the donors. In addition, we have calculated the sample size for an expected mean reduction of 25% and a reduction of 50% after a putative chemical exposure. It is obvious that in addition to donors the variability also depends on the markers analyzed. Some of them are very robust and give consistent results even with a small sample size (highlighted in grey, **Supplementary Table 9**) as for example all markers in the MAT (MAIT cell activation test) or BLAT (B cell activation test), whereas others such as IFNg in CD4 cells or NKT cells are less robust. Unless otherwise noted below, baseline levels of the analyzed activation markers in unstimulated samples were very low and are not shown.

### T Lymphocyte activation test (TLAT)

In the TLAT assay we attempted to obtain a broad lymphocytes response with a special focus on subtypes of T lymphocytes (**Figure 1**). As shown in **Figure 2** and in **Supplementary Figure 1** we have selected two activation markers (CD69 and CD154), cytokines (IFN-γ and TNF) and degranulation marker CD107a to be able to estimate the immune response upon anti-CD3 stimulation. The extent of immune response to aCD3 stimulation differs in magnitude and is cell type specific. As shown in tSNE analysis (**Figure 2**), the cytokines IFN-γ and TNF are strongest secreted in CD8+ cells whereas CD107a is expressed on many lymphocyte types including B, NK, CD4+ and CD8+ cells. CD69 is also widely distributed on NK, CD4+ and CD8+ cells. CD154 is mostly expressed in activated CD4+ cells and to a minor extend on CD8+ cells (**Figure 2A**). Next, we focused on maturation markers and we observed that IFN-γ and TNF are mostly secreted by effector memory (EM) CD8+ cells. CD107a is expressed on EM CD8+ cells, translational memory (TM) CD8+ cells and TM CD4+ cells, and to a lesser extent on naïve CD4+ cells. CD69 is expressed mostly on TM, central memory (CM) and naïve CD4+ cells, and on TM and EM CD8+ cells. CD154 activation marker is expressed on EM, naïve, TM and CM CD4+ cells as well as on TM and EM CD8+ cells (**Figure 2B**). In the case of Th subtypes, IFN-γ was less secreted by Th1 cells and to some extend by Th2 cells and by Tfh cells. TNF was also secreted by Th1 cells, by Th2 cells and also detectable in Th22 cells. CD107a was expressed mainly on Th1 cells, highly on Th22 cells, on Tfh cell subtypes (mainly on Th1, Th1/17 and Th17 subtypes) and to a lesser extent on Th2 cells. CD69 was expressed on Th1 cells and Th1/17 Tfh cells and partially also on Th2 cells. CD154 was distributed similarly to CD69 (**Figure 2C**). The detailed expression of activation markers is presented in **Supplementary Figure 1**. As shown in this figure, the level of activation marker expression is different on different cell subtypes, allowing the choice of the marker that best describes the immune response (e.g. CD107a on CD8+ EM or CD154 on CD4+ EM cells). Depending on the cell type and marker, the response to stimulation with anti-CD3 antibodies is up to 40%, e.g. (mean ± SD) 36.8 ± 10 for CD107a+CD8+ cells, **Supplementary Table 9**. It can also be seen that in the case of B and NK cells, there is hardly any change in the analyzed parameters even after 6 hours of stimulation with aCD3 antibodies. This suggests that the markers selected for T-cell analysis are T-cell specific and that this is a correct approach to using anti-CD3 antibodies to target the T-cell response.

### MAIT cell activation test (MAT)

To activate MAIT cells we used a stimulus specific for these cells, the bacterial strain *E. coli* K12. Stimulation at 10 bacteria per cell (BpC) has previously been validated (14) as the correct ratio to give optimal results for IFN-γ and TNF production in these cells. After this stimulation, 48.8% ± 6.8 of MAIT cells produced TNF and 42.5% ± 11.2 of MAIT cells produced IFN-γ, **Supplementary Table 9**. The percentage of MAIT cells expressing CD69 after this stimulation was very high (90% ± 7.1). This would not allow the assessment of an increase in the expression of this marker after chemical exposure, indicating that CD69 is not the optimal activation marker in this case. There was no detectable expression of the activation markers in unstimulated cells (representatively shown in **Figure 3**).

### Basophil activation test (BAT)

In our test, basophil activation was estimated based on the expression of the activation marker CD63. We observed a low baseline expression of CD63 in unstimulated basophils (up to 5%), **Figure 4**. Activation after aFcϵR1 stimulation was induced in about 40% of basophils, **Supplementary Table 9**. In the case of fMLP stimulation, the induction was seen in 42.3% ± 19.8 of basophils (**Figure 4**). Of note is our observation that the responses to the selected stimuli are very donor dependent and can even exceed 80% of activated cells in the case of fMLP and over 50% for aFcϵR1 stimulation (**Supplementary Figure 2**, **Supplementary Table 9**). Therefore, we recommend that the concentrations of these stimulants should be tested for each individual donor before performing the immunomodulatory tests with chemicals.

### Cytokine activation test (CAT)

In the cytokine activation assay, the cytokines IL-12/15/18, selected to mimic chronic stimulation, induced the activation of several cell types including MAIT cells, γδT cells, NKT cells and NK cells (**Figure 5A**). The selected markers, the cytokine IFN-γ, the activation markers CD69, CD137 and the degranulation marker Granzyme B showed a cell type specific activation pattern. In MAIT cells, the CD69 expression to cytokine stimulation was the strongest (68.1% ± 18.9), followed by Granzyme B expression (26.9% ± 12.5), IFN-γ expression (30.9% ± 12.6) and, to a lesser extent, CD137 expression (3.8% ± 1.6) (**Supplementary Table 9**, **Figure 5B**). No background expression of these markers was observed in unstimulated MAIT cells. In the case of NK cells, background expression of Granzyme B was at a high level (37% ± 16.6) in unstimulated samples, data not shown. However, after cytokine stimulation, Granzyme B expression was induced to a higher level, reaching 51% ± 3.3 activation of NK cells, **Supplementary Table 9**. This increase in the percentage of activated cells was accompanied by an increase in mean fluorescence intensity (MFI), which could also be used as a readout of NK cell activation, data not shown. In contrast, the expression of CD137 (6.1% ± 4.3) and IFN-γ (17.3% ± 12.2) in activated NK cells was low to moderate (**Figure 5C**, **Supplementary Table 9**). In γδT cells, Granzyme B expression was also high in unstimulated cells (15.7%% ± 10.4), data not shown. Again, after stimulation, the percentage of these stimulated γδT cells increased (43.9% ± 13.6, **Supplementary Table 9**), with a notable MFI shift. Regarding the expression of IFN-γ, CD69 and CD137 in γδT cells after stimulation, the percentages of activated cells were 37.3% ± 20.3, 56% ± 20.1 and 6.2% ± 3.2, respectively (**Supplementary Table 9**, **Figure 5D**). For NKT cells, the number of activated cells expressing Granzyme B could be induced up to 42.1% ± 6.2 by cytokine stimulation. Excepting for Granzyme B, there was no baseline expression of activation markers in NKT cells in the unstimulated samples (data not shown). Cytokine stimulation induced the expression of CD69 in 31.9% ± 16.9, IFN-γ in 12.1% ± 9 and CD137 in 4.9% ± 2.4 of NKT cells (**Supplementary Table 9**, **Figure 5E**).

### B lymphocyte activation test (BLAT)

In the B cell activation test, we identified B cells staining for CD19 and CD20 and additionally naïve B cells via IgD and CD27 specific antibodies (**Figure 6A**). The activation markers CD69 and CD83 were chosen to measure the activation after B cell-specific stimulation with anti-IgG/IgM, R848, aCD40 and CpG ODN2006. Both, the % of activated cells and the shift in MFI were used to identify CD69 and CD83-expressing B cells (**Figure 6B**). Both markers were also expressed on unstimulated B cells. Nevertheless, an increase in CD69 and CD83 expression was observed after stimulation, which was only visible at the MFI level. The MFI of CD83+CD19+ cells increased from 29930 ± 5627 (unstimulated) to 36733 ± 5265 (CpG ODN2006 stimulated), 101992 ± 17398 (R848 stimulated), 66816 ± 13418 (anti-IgG/IgM stimulated) and 50560 ± 22038 (anti-CD40 stimulated), **Supplementary Table 9**. For CD69, the effects were less pronounced, **Supplementary Table 9**. CD20 was shown to be more stable expressed on B cells upon stimulation with anti-CD40 antibodies (see shift of CD19 marker upon aCD40 Abs stimulation in **Figure 6B**). Cells with high expression of CD20 and lower expression of CD19 were low abundant and responded less to stimulation that is why we did not focus on their activation pattern. Expression of activation markers on CD20 positive and naïve CD19 positive cells is shown in **Supplementary Figure 3**.

### NK cell activation test (NKAT)

NK cells were defined as CD3- and CD56+ cells (total) and further described with the markers HLA-DR and CD16 into early, mature and terminal NK cells as shown in **Figure 7A**. The K562 cell line was chosen as a specific stimulus for NK cell activation. In total NK cells the two selected activation markers, CD107a and CD137, were highest expressed, 34.8% ± 6.1 and 29.8% ± 10.4 respectively, when PBMCs were stimulated with K562 at a ratio of K562:PBMC (1:6.125), **Figure 7B**, **Supplementary Table 9**. A similar percentage of activated cells expressing these markers has been observed in the early, mature and terminal NK cell subpopulations, **Supplementary Figure 4**. As representatively shown in **Figure 7B** the percentage of NK cells double positive for these markers is lower than the percentage of cells expressing only one of the markers, indicating differences in the NK response to K562 cells with respect to the expression of CD107a and CD137 (**Figure 7B**).

### Effect of immunomodulatory substances

As a proof of concept, the effects of LPS, rapamycin and cyclosporin A were tested for their immunoactivating and -suppressing activity in the TLAT, MAT, CAT, BLAT and NKAT assays (**Supplementary Figures 5**-**10**). With cyclosporine A we observed a downregulation of immune responses in CD154+CD4+, IFNg+CD4+ and TNF+CD4+ cells, but not in CD69+CD4+ and CD107a+CD4+ cells (**Supplementary Figure 5**). With regard to CD8+ cells, cells expressing CD154, IFNg or TNF also responded strongly to CsA, but in addition also CD69-expressing cells (**Supplementary Figure 6**). We observed that rapamycin and LPS were not suitable modulators in the TLAT assay. In the MAIT cell activation test (MAT), downregulation of activation was stronger with CsA compared to rapamycin (**Supplementary Figure 7**). LPS did not exceed a stimulatory effect, but rather a downregulatory effect (**Supplementary Figure 7**). In the cytokine activation test (CAT), rapamycin, but not CsA, downregulated the immune response, especially in IFNg-producing cells (**Supplementary Figure 8**). In the BLAT assay, a modulatory effect of the substances used was observed at the MFI level. LPS increased the expression of CD69 in all stimulated CD19+ cells (**Supplementary Figure 9**). As expected, the strongest effect of LPS was observed on NK cells in the NKAT assay. In particular, a modulatory effect of LPS and CsA was observed in CD137+ NK cells (**Supplementary Figure 10**). CsA specifically inhibited the activation of CD137-expressing cells, but only to a lesser extent the activation of CD107a-expressing cells.

## Discussion

In this paper we propose a battery of *in vitro* assays for immunotoxicology to be considered for the safety assessment of new chemicals. The assays have been designed to cover most of the immune cell subpopulations found in human blood. The main advantage of these assays is that they are simple, rapid, sensitive and reproducible. They are based on human primary blood cells and represent the natural proportion of cells found in human blood. The use of flow cytometry allows to study the effects of chemicals at the level of individual cells. We have also chosen to use a range of cell type-specific stimuli to stimulate the target cells (such as CD3 antibodies for T cells or *E.coli* for MAIT cells), which allows us to validate the extent to which the effects on the target cells can be influenced by a tested chemical. If the aim is to assess immunostimulation and suppression simultaneously, titration of the stimulus must be performed *a priori* in order to determine the concentration that allows both an increase and a decrease in the immune response.

### T lymphocytes

Here, we attempted to create a multi-color cytometric panel describing the majority of T cell subsets. In this assay we stimulated the cells by CD3 specific antibodies. We applied a wide range of T cell phenotype describing parameters and we used 5 activation parameters including cytokines, activation markers and degranulation molecules. In a similar assay, but with a simplified multicolor panel and by using CD3/CD28 stimulation we already analyzed the effect of PFAS on lymphocytes (14). The present assay has the advantage of identifying a greater number of T cell subtypes with less stimulatory agents.

There are already some established assays for the analysis of lymphocytes in immunotox assays. These include first of all Mishell-Dutton (MS) assay which originally was designed to measure proliferation of mouse lymphocytes from spleen in response to sheep erythrocytes (15). This assay was further developed for rat PBMCs (16) and finally for human PBMCs with adaptation of a test to influenza antigen-specific T cells response (HuLA – Human Lymphocytes Activation Assay) which involves the proliferation of T and B cells as well as estimation of antibody secretion by B cells (17, 18). Other tests are focusing on peripheral blood mononuclear cells in depth characterization including cell signaling estimation by phosphorylation of some key molecules involved in immune cells cell signaling so called Single Cell Network Profiling (SCNP) (19, 20). Similar to our approach with multiparameter flow cytometry, others attempted to establish complex assays for multiplex immune cell characterization by mass cytometry (MC) (21). This methodology allows for deep immune phenotyping, but has the disadvantage of being more expensive. The methods like SNCP or MC are to laborious and expensive to be used for routine chemical testing and may serve only as a follow-up in depth testing of mechanism of selected chemicals. In addition to the use of primary human immune cells, there are increasing attempts to establish rodent and human cell lines for chemical testing to avoid the use of living donors. For example, Jurkat T cell line has been used for chemical testing in reporter gene assays (22), at the level of gene expression by PCR (23) and for phosphorylation of intracellular proteins (24). However, these malignant cells are not the true equivalents of primary cells, neither in their phenotypic markers nor in their functionality. Further, immune cells interact within networks and require contact to other immune cell subpopulations to exert their function, a feature that cannot always be mimicked *in vitro*. Activation of T lymphocytes has also been analyzed based on cytokine secretion in supernatant from PBMCs or direct from the cells in the whole blood by using ELISA (25, 26). Also human lymphoblastoid cell line was used to assess cytokine production for assessment of four immunotoxic compounds; tributyltin chloride, cyclosporine A, benzo(a)pyrene and verapamil hydrochloride (27). This however was performed by ELISA and the cells were unspecifically stimulated with PMA/ionomycin. These assays involving cytokines present in the supernatants greatly differs from the established TLAT assay reported here where each immune cell type can be analyzed separately based on profound cell immunophenotyping.

The proposed T lymphocyte activation test covers also Th subsets and maturation stages of T cells (28) which help to identify the T cell subtypes which are affected by chemicals upon T cell specific stimulation. Furthermore, in the cytokine activation test, we focused on characterization of NK cells and innate-like T cells: NKT and MAIT, γδT cells in response to cytokines IL-12/15/18. All these cells act in innate immune response and the selected cytokines mimic chronic exposure during immune response (29, 30) which leads to other cytokines release (like IFN-γ or TNF) which takes place at tissue side and may lead to organ failure (31).

In addition, our assay is fast and easy to perform, compared to others which last from 2 days (cytokine release), 4 days for Th cells differentiation up to 7d for cell proliferation (32).

### NK cells

NK cells are representatives of innate immune response and numerous *in vitro* tests have been already established to analyze their activity. These methods employ K562 (human erythroleukemia cell line) cells which are either a target of NK cells lytic activity (33, 34) or may be used as stimulators to induce NK response which is evaluated by appearance of degranulation marker CD107a (35, 36). Also proliferation of NK cells was used by others for chemical testing (37). In the NK cell activation assay introduced here, we combined the use of K562 cells as stimulators and two NK cell activation markers, CD107a and CD137. The latter in particular was found to be highly expressed on activated NK cells. The use of both activation markers proved to be very sensitive and led to the description of CD107a only expressing NK cells, CD137 only expressing and double positive for both markers NK cells. In our cytokine activation assay, NK cell activation was assessed by IFN-γ secretion and expression of CD69, CD137 and Granzyme B. As NK cells express high baseline levels of Granzyme B, this marker was not found to be suitable for assessing NK cell activation.

### MAIT cells

MAIT cells are the cells orchestrating the immune response to pathogens on the mucosal cell surface (38). They respond to bacterial metabolites of vitamin B origin upon activation of TCR receptor via antigens presented by the MR1 molecule with the subsequent induction of proinflammatory cytokines like TNF or IFN-γ (39). The method described here can be used for the evaluation of MAIT cell responses upon stimulation with *E.coli.* We already used a similar method for the evaluation of chemical effects on these cells for bisphenols, PFAS and samples from wastewater treatment plants (WWTP) (13, 14, 40). The other assay we propose, the cytokine activation of MAIT cells via mixture of IL-12/15/18, serves as surrogate for antigen-independent MAIT cells activation which mimics innate/adaptive immune response leading to chronic activation (41). In the mentioned cytokine activation assay we proved not only that MAIT cells are able to respond to this cytokine cocktail but also that NK, NKT and γδT cells do it too, hence more cell types are targeted highlighting the strength and suitability of the proposed flow cytometry assay for multi-parameter marker selection.

### B cells

Characterization of the B lymphocyte response can now be measured on the basis of cell proliferation (17), antibody production, cytokine secretion or at the level of surface marker expression (42). There are also attempts to employ B cell lines like Namalwa B cells for immunotoxicity testing which was shown for PFAS (43). In general there is a high correlation between B cell specific responses (like immunoglobulin secretion and cytokine production) and surface marker expression upon stimulation (42, 44). Here, we attempted to characterize B cell responses by analyzing surface activation markers in PBMC after stimulation with specific agents. We have selected CD69 and CD83 as the most sensitive activation markers. When establishing the tests presented here, we also tested other activation markers like CD70, CD80, CD86 but found them not to be suitable for the characterization of B cell activation (data not shown). Regarding the selected stimuli, by choosing R848 and CpG ODN2006 we were able to determine the innate immune response induced via TLR7/8 and TLR9, respectively. On the other side, by selecting anti-CD40 and anti-IgG/IgM antibodies, we were able to induce the adaptive response of B cells via CD40 and BCR receptor, respectively (42). Hence, by selecting different stimuli, we are able to estimate the effects of chemicals by modulation of innate and adaptive B cells response.

### Basophils

Basophils are known to be involved in allergy through the release of IL-4 and their ability to drive antibody responses. This cells play a central role in the regulation of the immune response, which is why their dysregulation by chronic or acute chemical exposure can lead to abnormalities in the immune response (45, 46). Especially their location on mucosal tissues and in the skin make these cells the key responders toward chemical exposure (47). We have selected basophils as proxy for innate immune response. In our assay, whole blood samples are stimulated in different ways to highlight the innate or the adaptive basophil response. We used fMLP, a chemotactic peptide being released by bacteria exerting innate immunity and anti-FcϵR1α which target the IgE receptor being involved in adaptive immune response. Thus, by selection of the stimulus we are able to discriminate between putative chemical effects on early and late immune responses. By application of our BAT assay we aim to estimate the effects of chemicals on hypersensitivity reactions (48, 49). We selected CD63 as basophil-specific activation marker (50) which correlates with the release of basophil mediators like cytokines and histamine (51). A similar approach for the evaluation of basophil activation in response to quinolones was proposed by Aranda et al. (52). The basophil assay presented has already been validated by us for PFAS, where we found a differential response of basophils to a PFAS mixture depending on the stimulus used (14).

### Limitations

The set of immunoassays presented for the testing of chemicals has its own limitations due to the nature of the human biological material, which, although obtained from healthy donors, may contribute to the variability of the response. The variability also depends on the marker analyzed. To overcome the variability, we recommend that the data following chemical stimulation be normalized for each marker to the values obtained with the activation stimulus only, as each donor may respond differently to the immune cell activation stimulus. The chemical effects are expressed as fold change relative to the corresponding control. This allows the focus to be on the modulation of this stimulation by the chemical exposure, rather than the individual variability of the response to the stimulant (14, 40). It is also a limitation that important immunomodulatory cytokines such as IL-10, IL-17 and TGF−β were not included in our tests because they are barely expressed after the short incubation times we used. Therefore, long-term tests will need to be established to account for chemical effects on these cytokines. We are aware that LPS is an innate, but not the right control to directly assess adaptive immune responses. In this respect, it may act more indirectly by stimulating innate immune responses. Thus, optimal activation and downregulation controls for each immune cell population remain to be evaluated and will be the subject of future research and method development. Another limitation may be that important immune cell subpopulations such as dendritic cells, monocytes or granulocytes were not considered. However, our aim in the present study was to provide fast immunological test systems. In the case of dendritic cells, complex culture conditions would be required as they need few days to differentiate from monocytes (53, 54). In the case of monocytes, the main limiting factor is that they tend to bind to the plastic surfaces, making it difficult to use these cells in flow cytometry. In this case, instead of using human blood monocytes, it is much more advisable to use monocytic cell lines such as THP-1 or U937 for chemical testing (55). Neutrophils could be added to the test portfolio and the effect of chemicals could be measured by the induction of reactive oxygen species after activation of these cells with fMLP or bacteria. However, caution must be exercised as the usability of this test is greatly affected by the rapid degradation of neutrophils, leading to unreliable results (56).

In addition, it is important to consider sex differences as they may influence the assay sensitivity. For example, endocrine disruptors may affect immune cells in a sex-specific manner due to differences in the expression of hormone receptors. The fact that we did not take the sex of the donors into account is a limitation of the present study. However, others have suggested using male donors for the initial testing and then confirming the effects in women at a similar stage of the menstrual cycle (57, 58). It is also possible that *in vitro* testing of chemicals may not reflect the real situation *in vivo*, where the chemicals are subject to metabolism that may either activate or inactivate them, depending on the mode of action (59). Taking all these limitations into account, data should be interpreted with caution and any potential effect of a chemical on the immune response can be verified by other available *in vitro* tests and, to a limited extent, by *in vivo* tests for selected chemicals to explain the mechanism of action.

## Conclusions

By selecting PBMC and whole blood as the biological material and addressing the analysis at the multicellular level, we have established a versatile test system for chemical effect testing. Our assays include T cells, NK cells, B cells, basophils and innate like T cells such as γδT, MAIT and NKT cells. By selecting specific stimuli and activation markers, the assays are suitable for identifying chemical effects on certain immune cell subtypes.

## Data availability statement

The original contributions presented in the study are included in the article/**Supplementary Material**. Further inquiries can be directed to the corresponding authors.

## Ethics statement

The studies involving humans were approved by Ethics Committees of the University of Leipzig. The studies were conducted in accordance with the local legislation and institutional requirements. The participants provided their written informed consent to participate in this study.

## Author contributions

GH: Conceptualization, Supervision, Writing – review & editing. AP: Conceptualization, Data curation, Investigation, Methodology, Visualization, Writing – original draft, Writing – review & editing. AZ: Funding acquisition, Resources, Writing – review & editing.
