# Supplementary material for: A comprehensive battery of flow cytometric immunoassays for the in vitro testing of chemical effects in human blood cells
Source: Front Immunol. 2024 Jan 2;14:1327960. doi: 10.3389/fimmu.2023.1327960 (PMC10790304; doi:10.3389/fimmu.2023.1327960)
Supplement: Supplementary file 1 [file DataSheet_1.docx]

**Supplementary Material**

**Expression of activation markers on the analysed T cell subtypes**

**A. CD4+ lymphocytes**


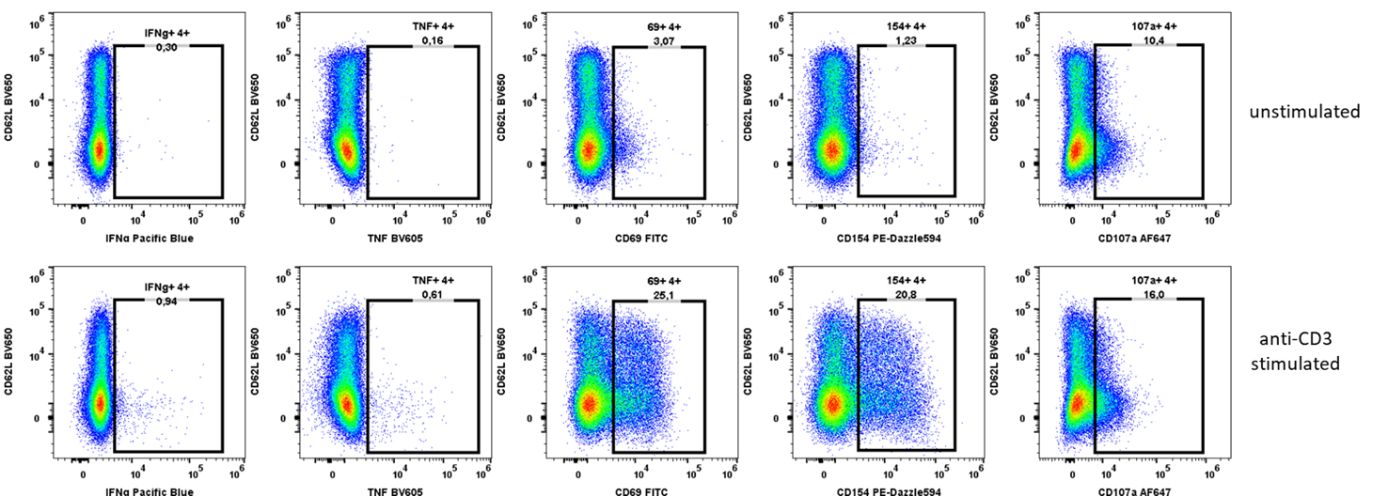


**B. CD8+ lymphocytes**


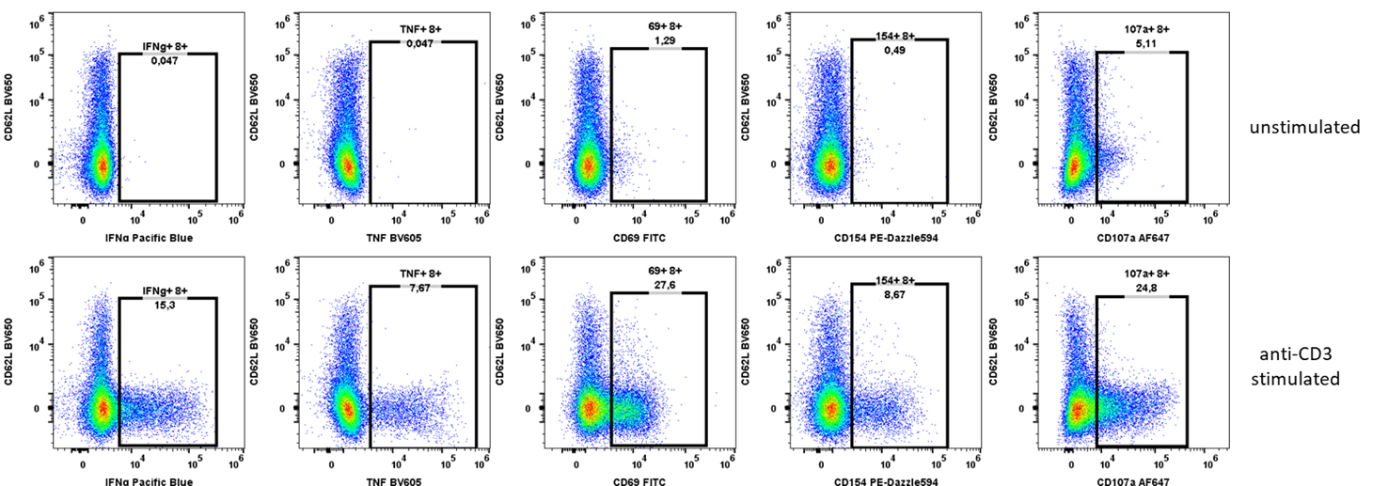


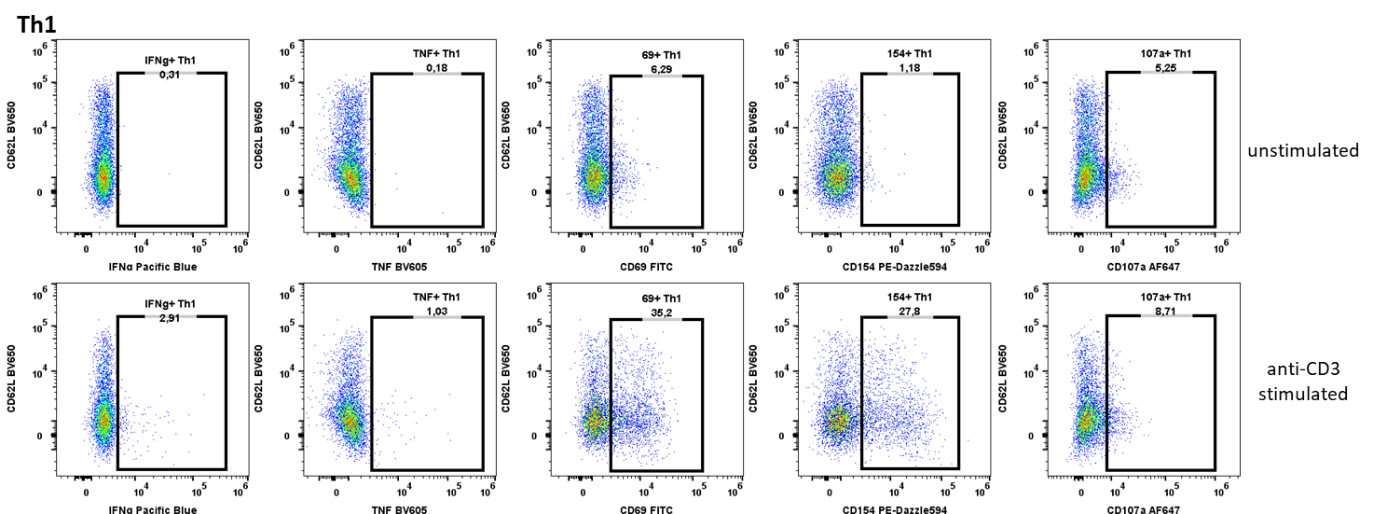


**C. Th cell subsets**


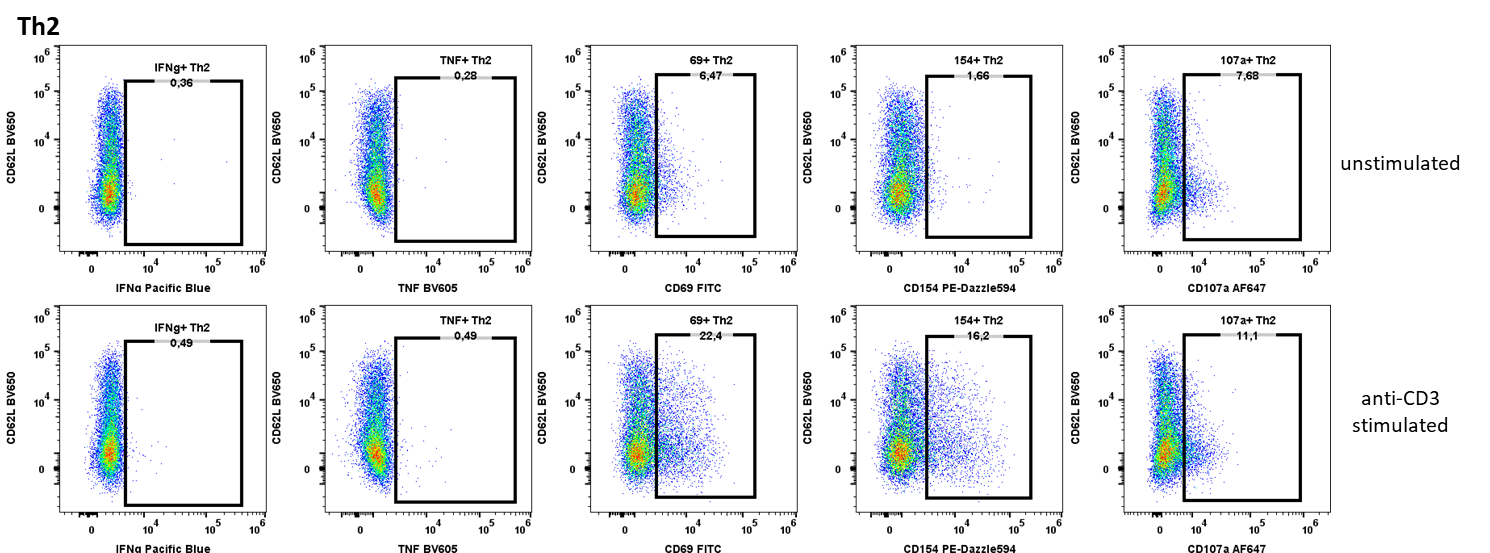

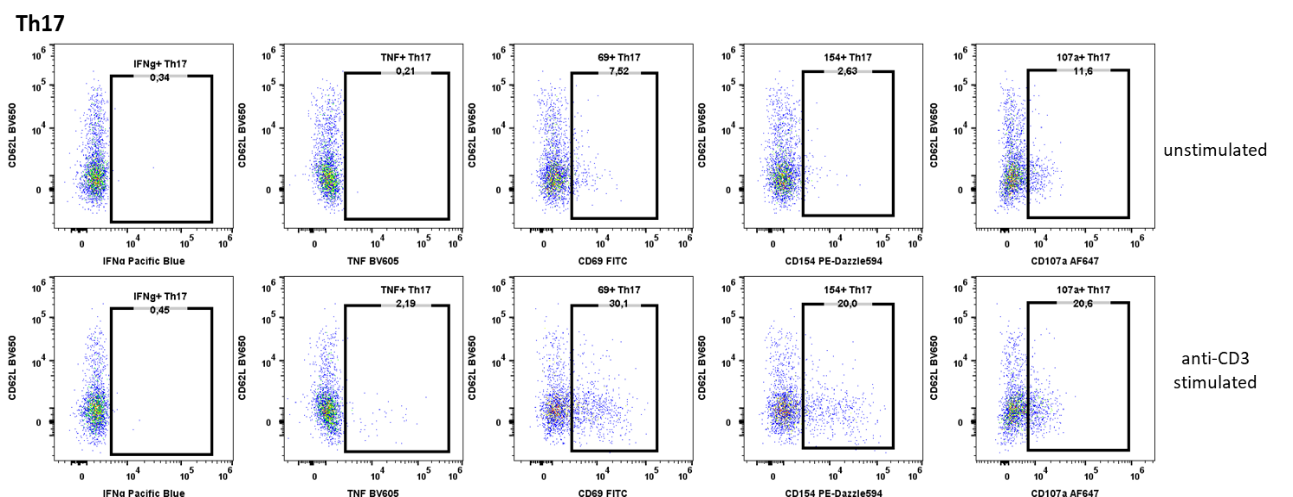

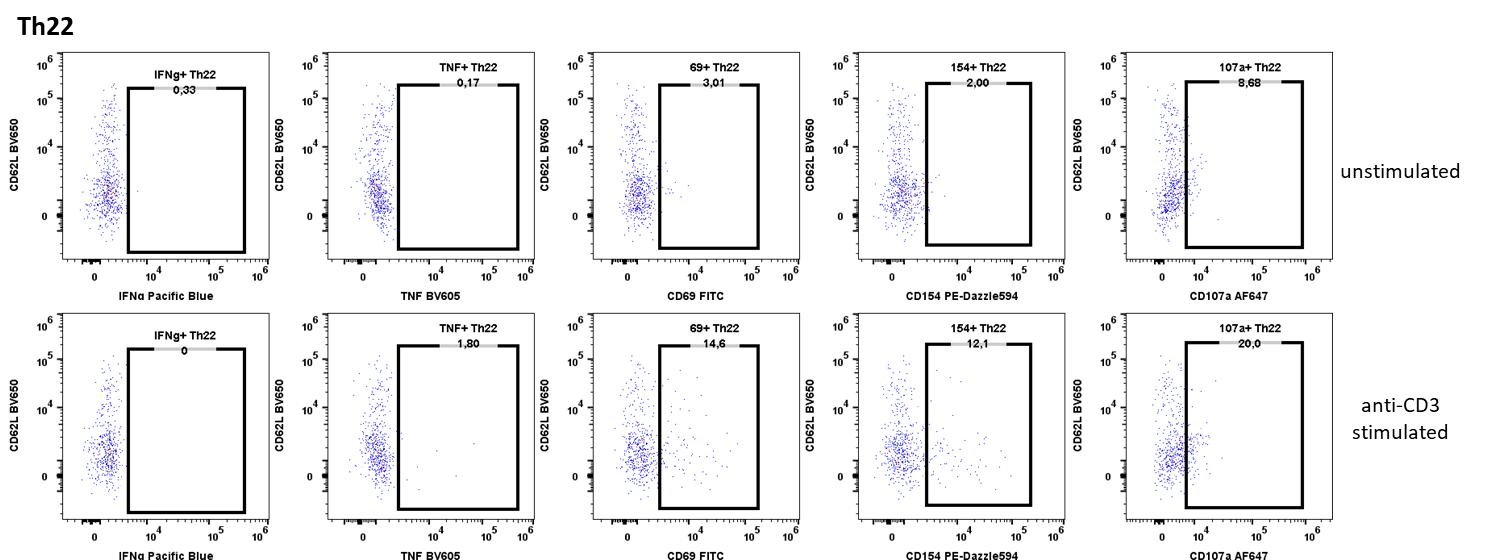

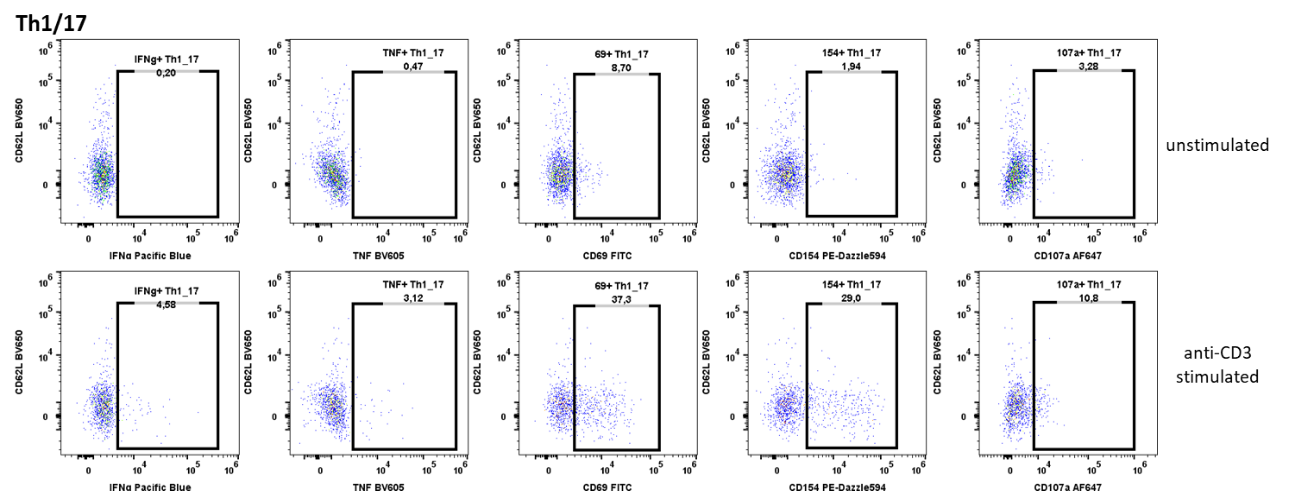


**D. Tfh cells subtypes**


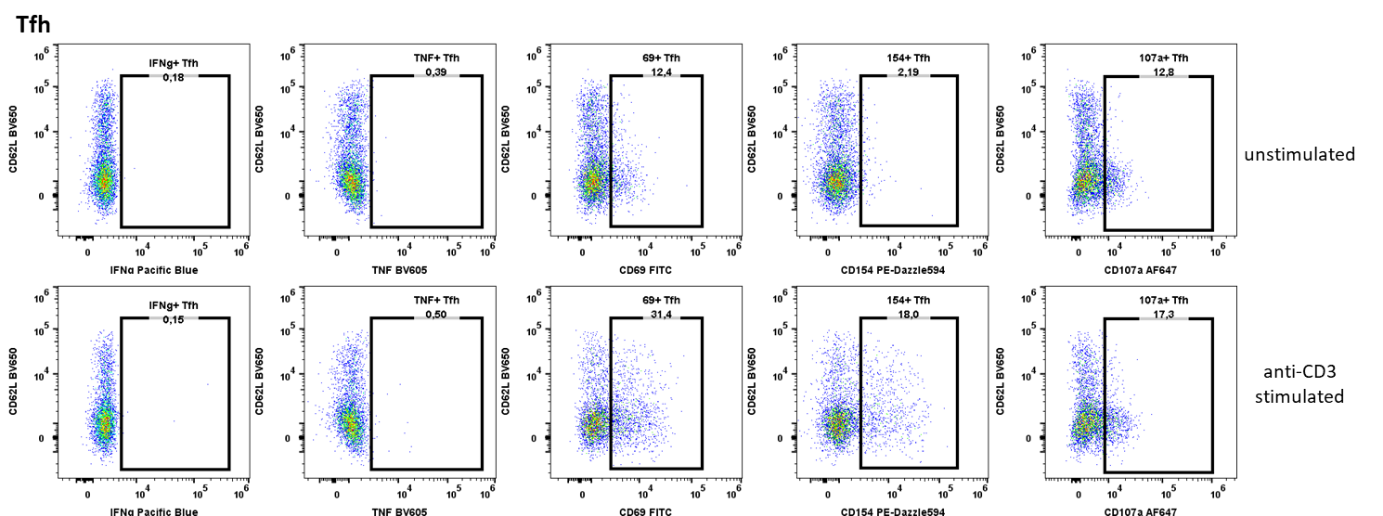

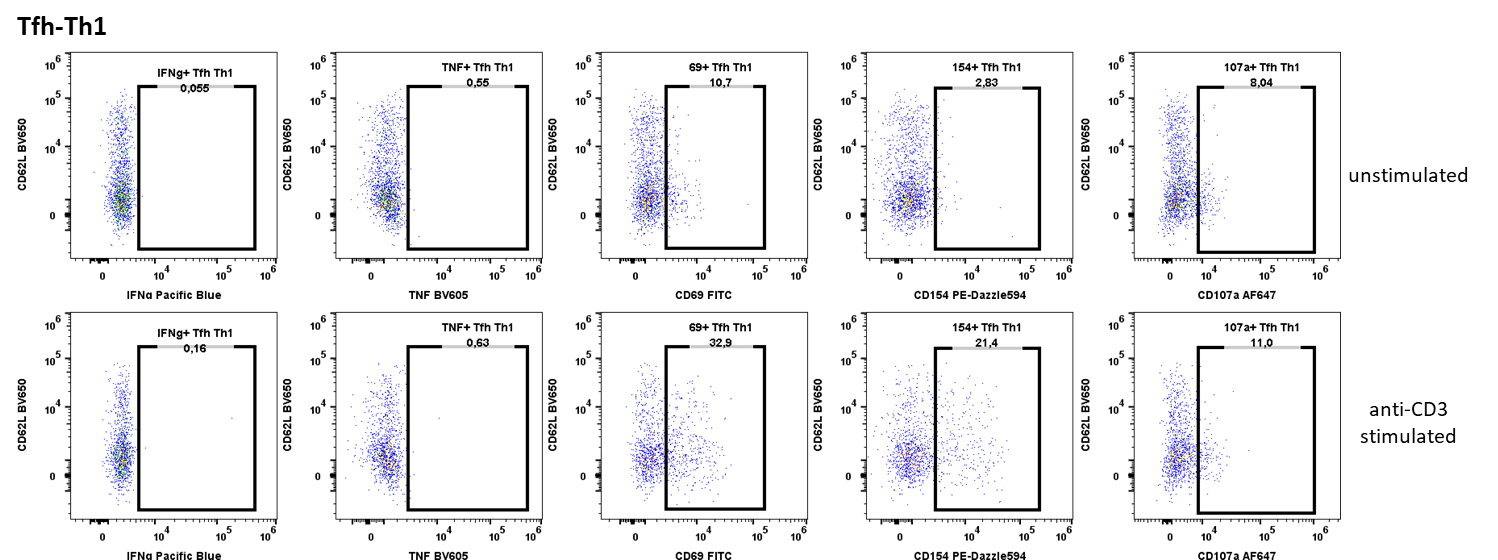

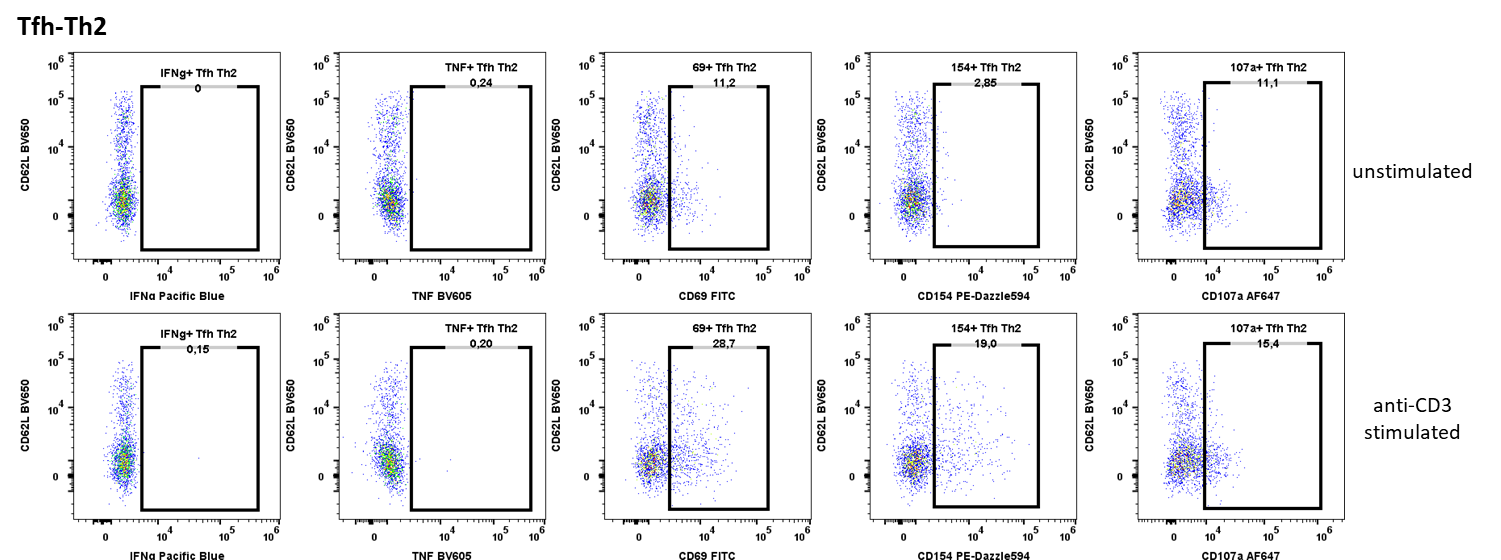

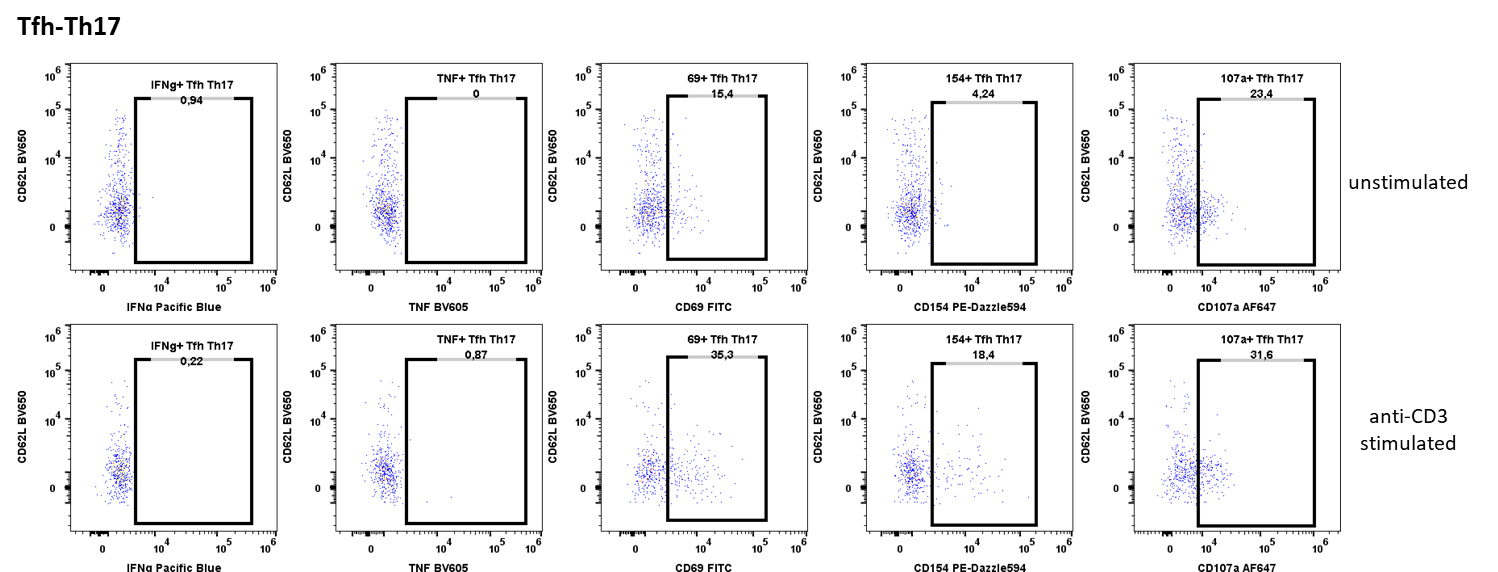

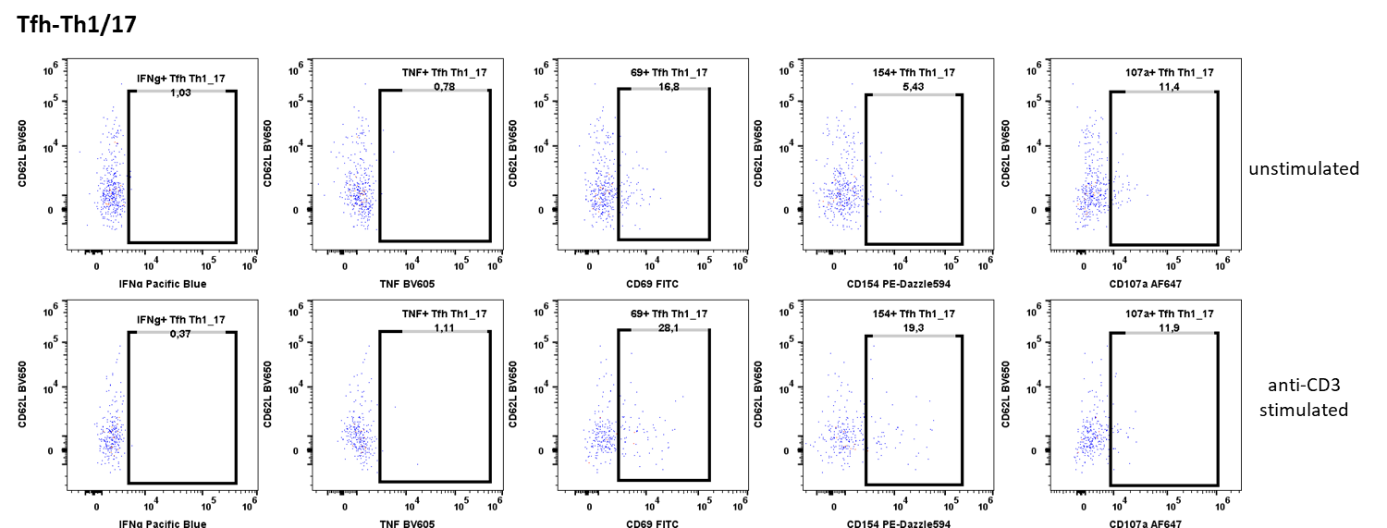


**E. CD4+ maturation subsets**


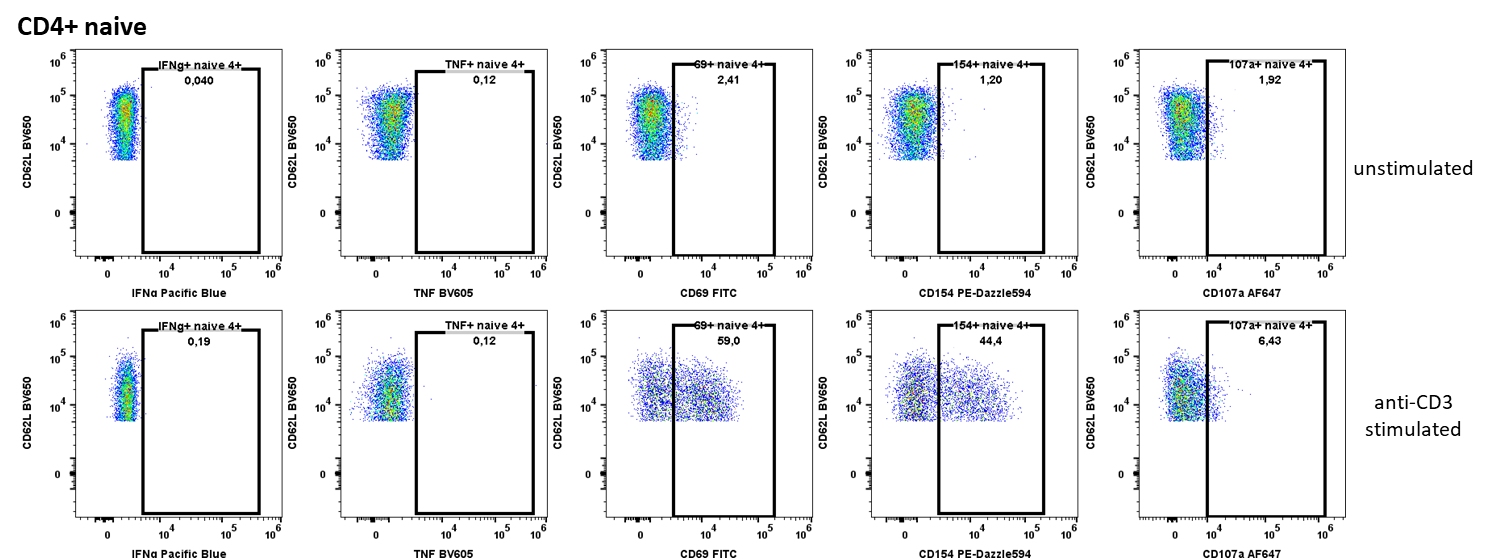

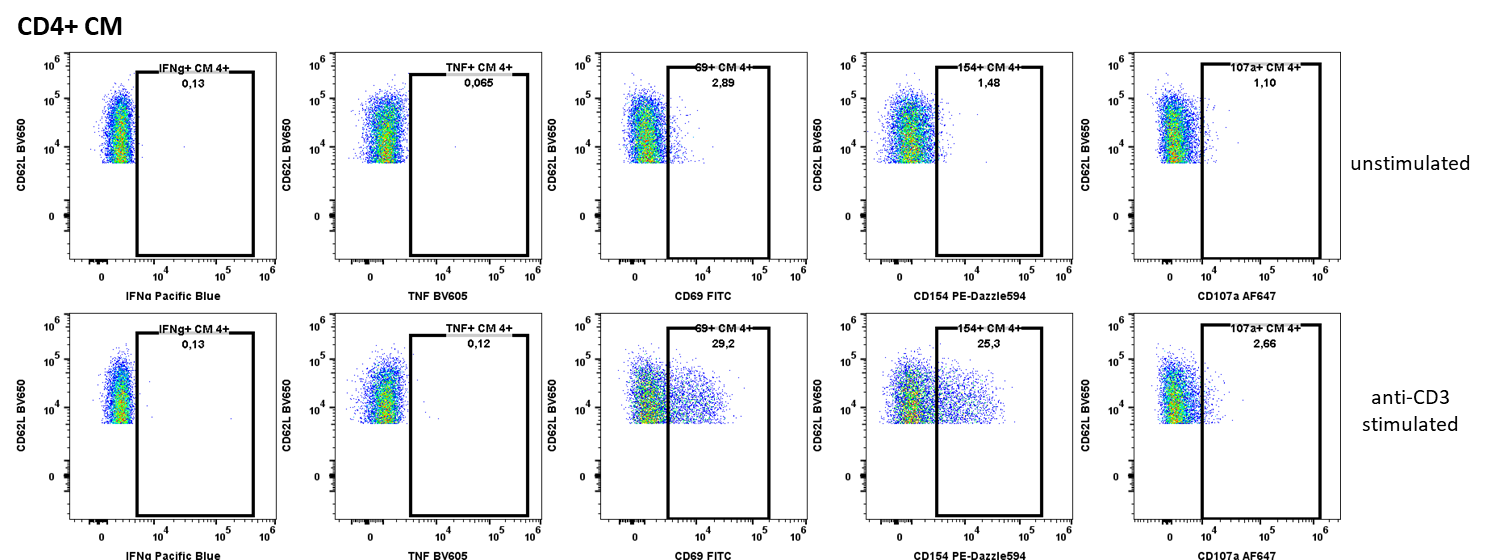

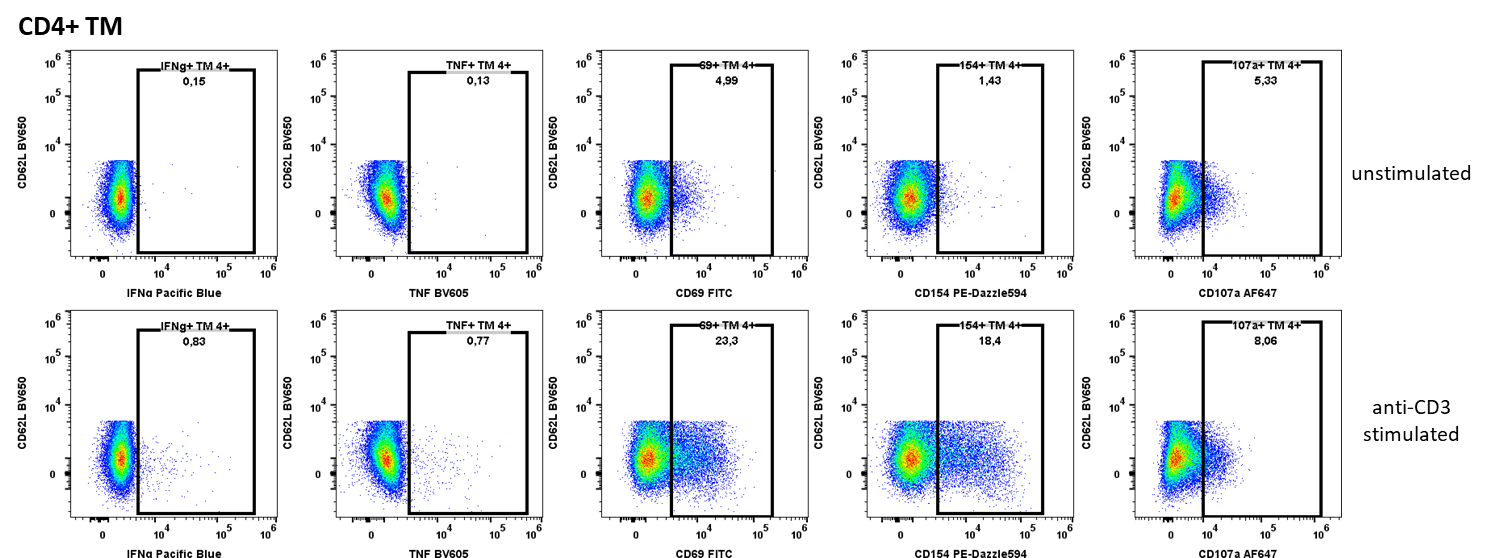

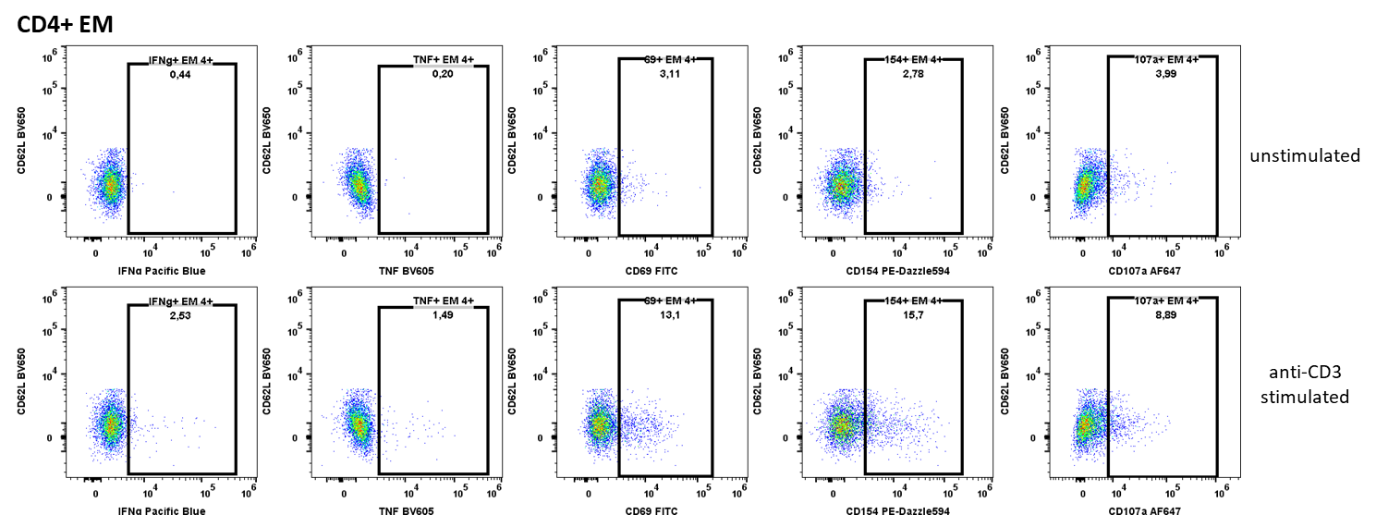

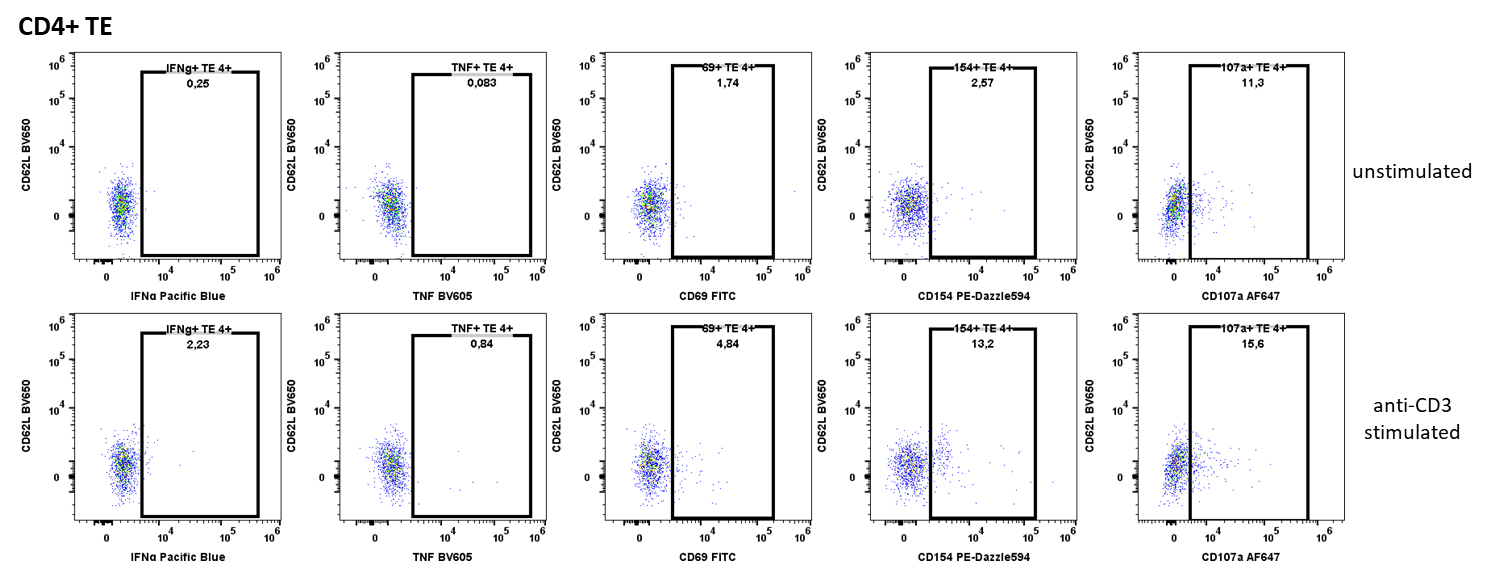


**F. CD8+ maturation subsets**


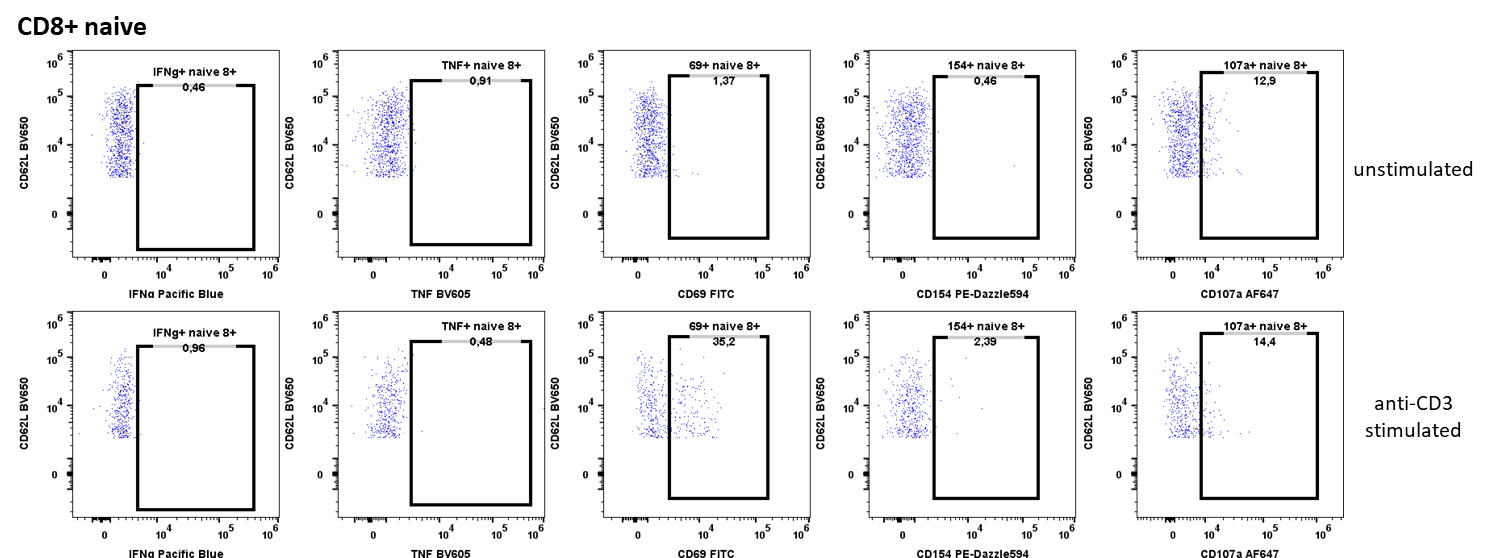

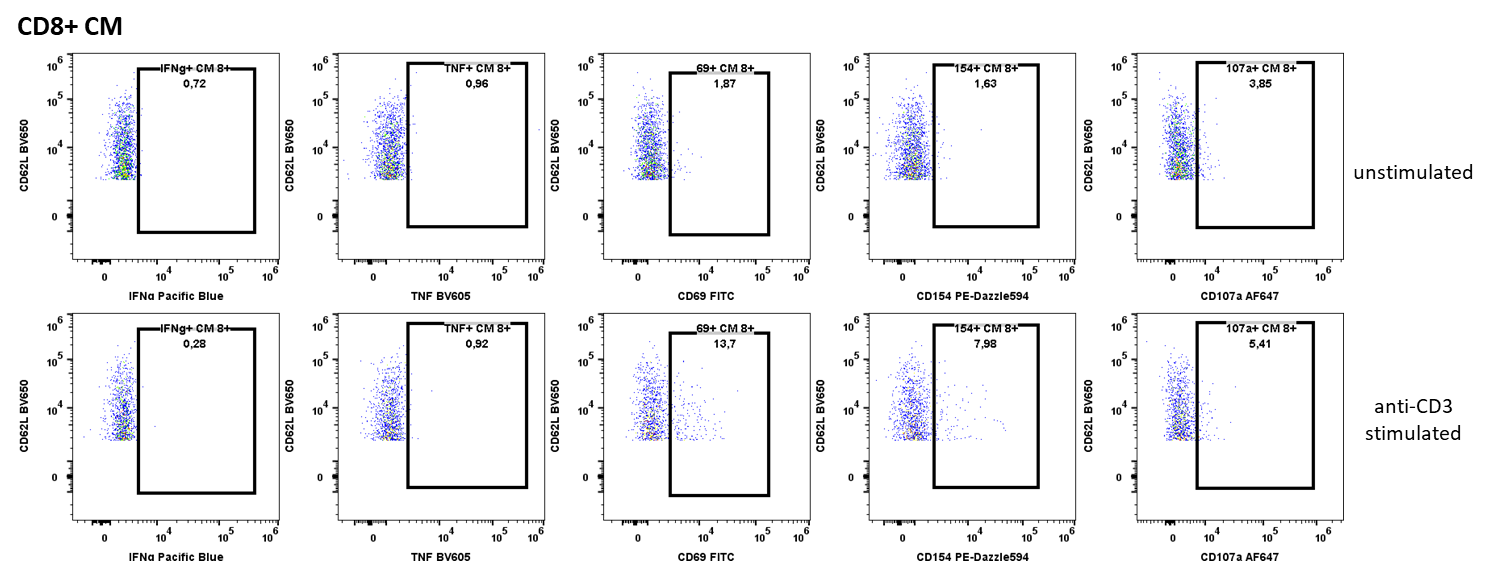

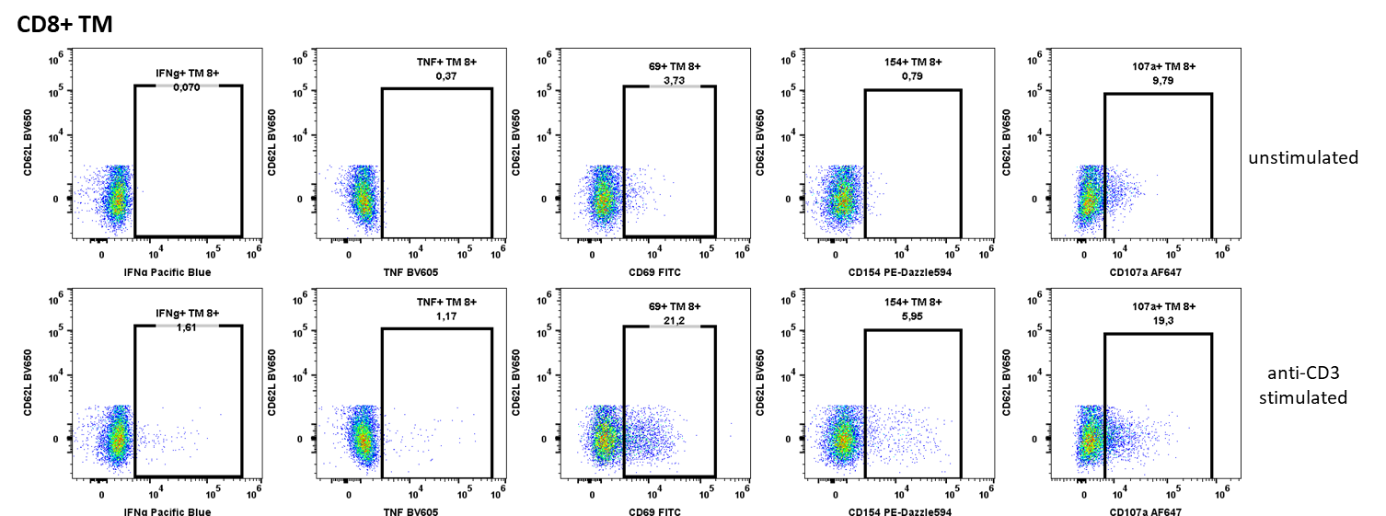

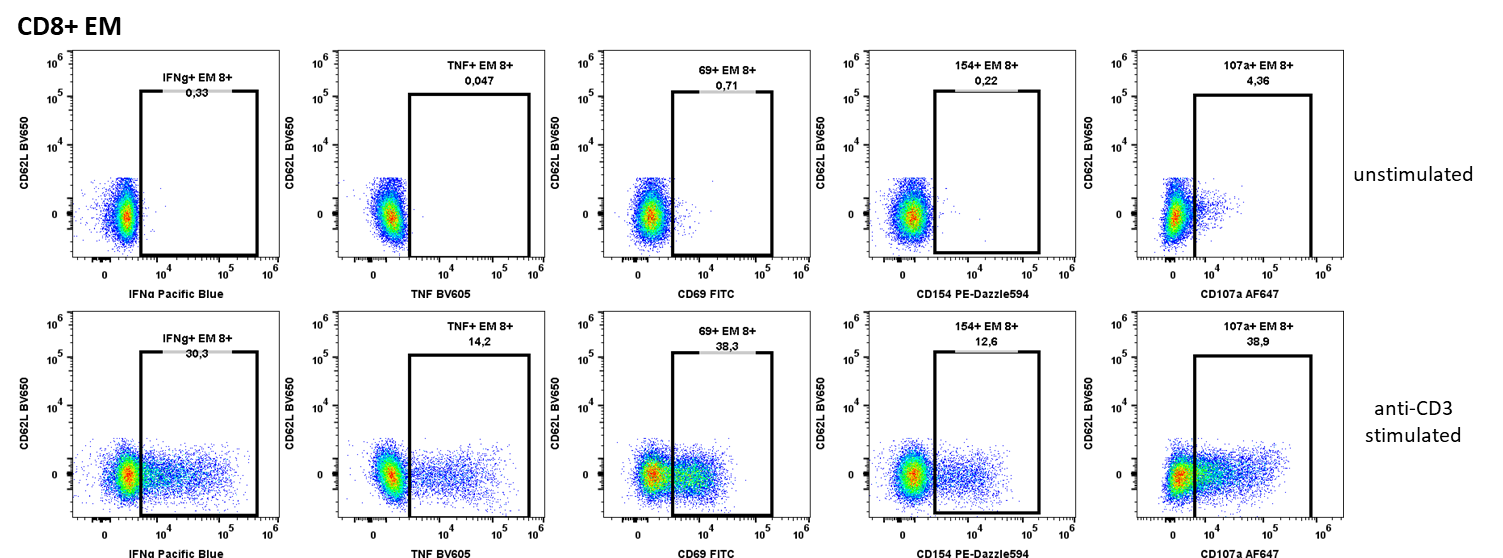

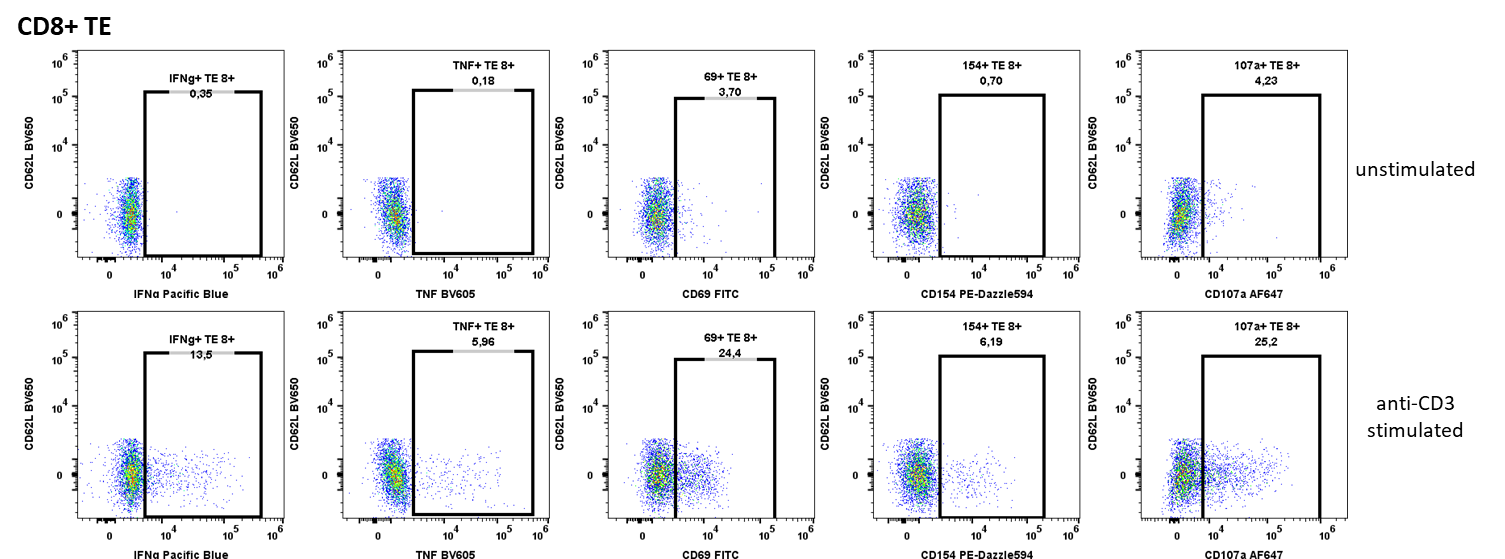


**G. Other lymphocytes**


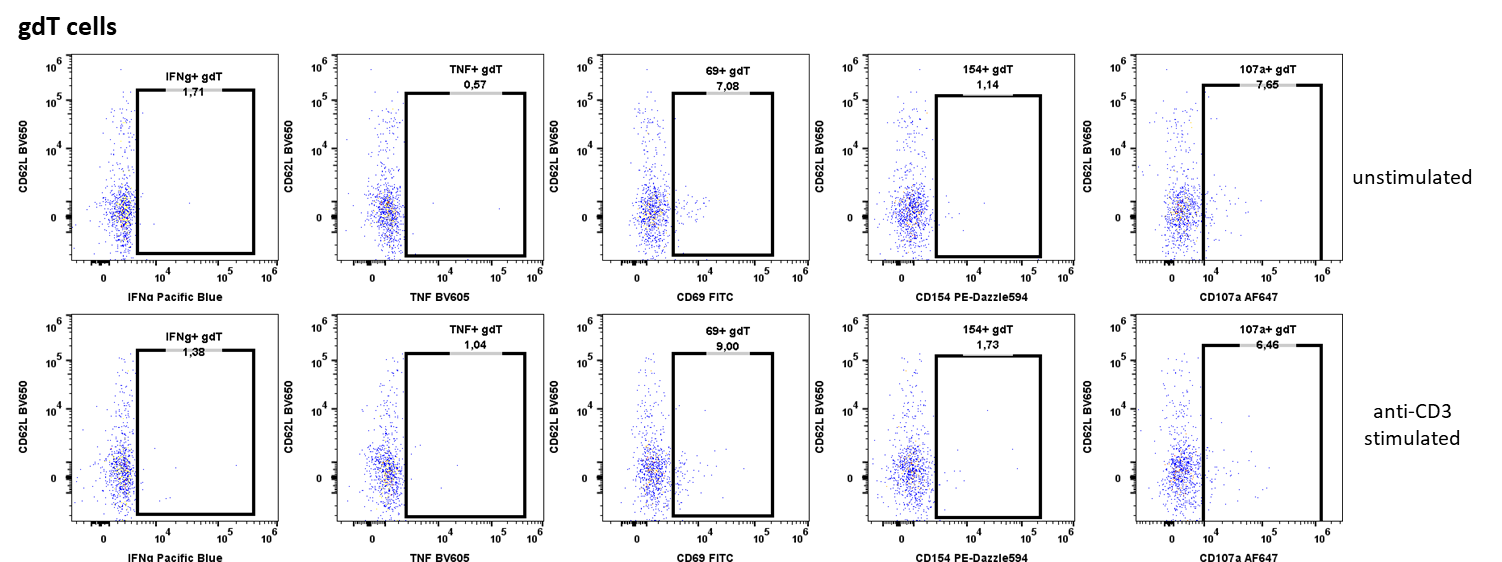

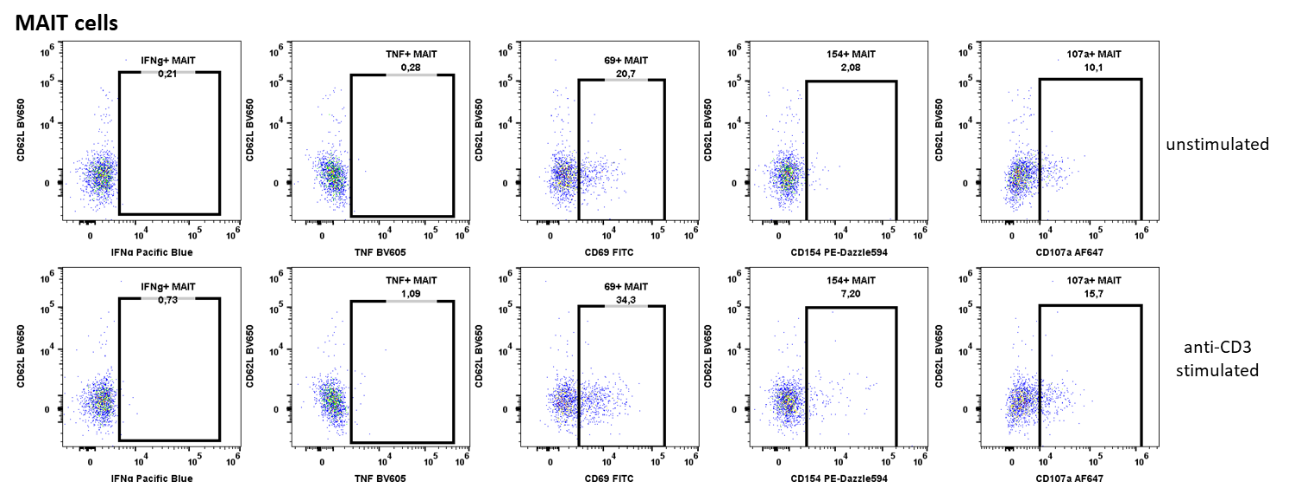

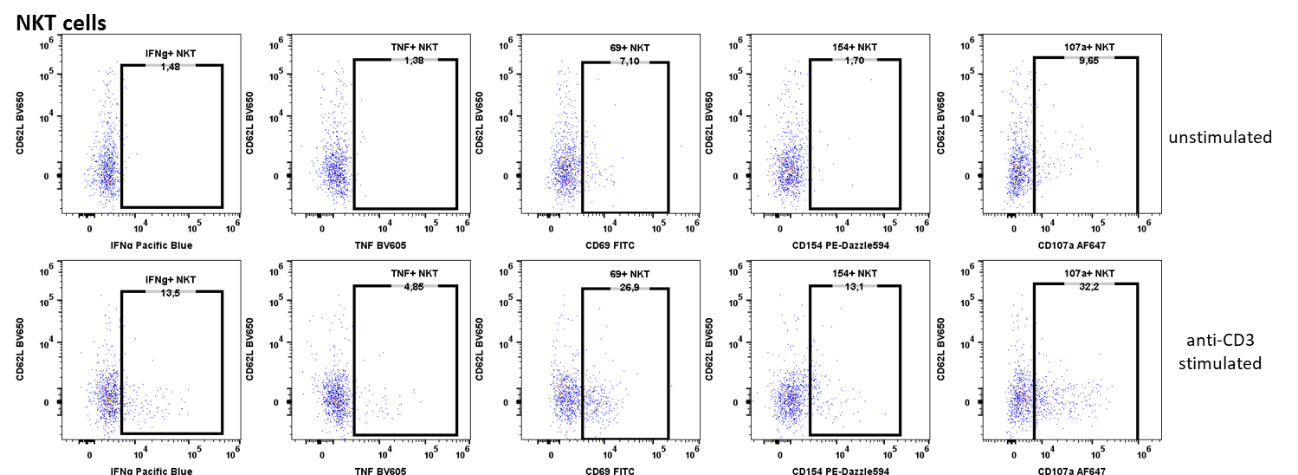

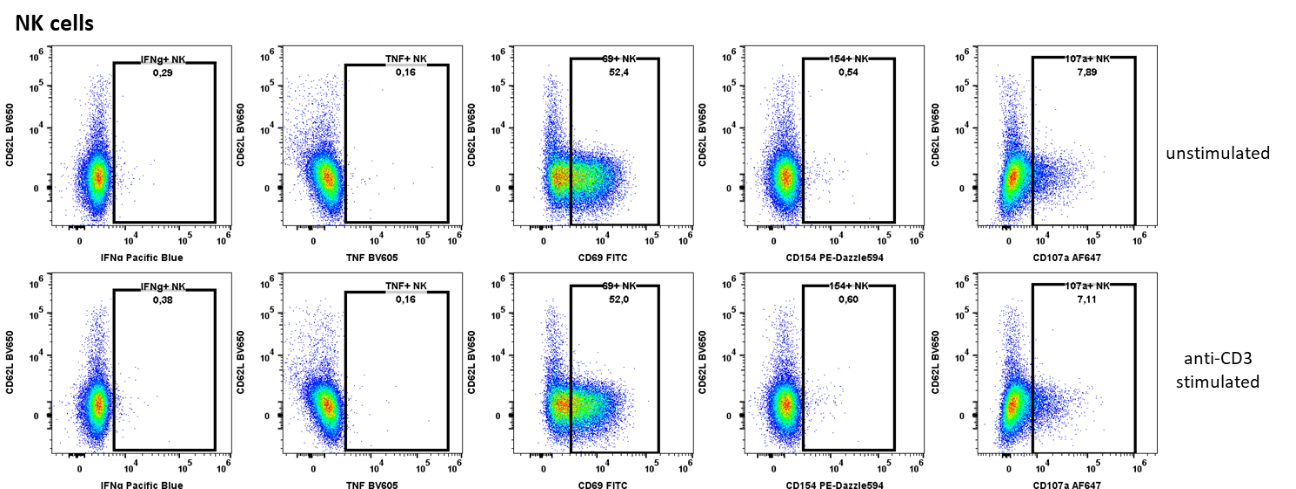

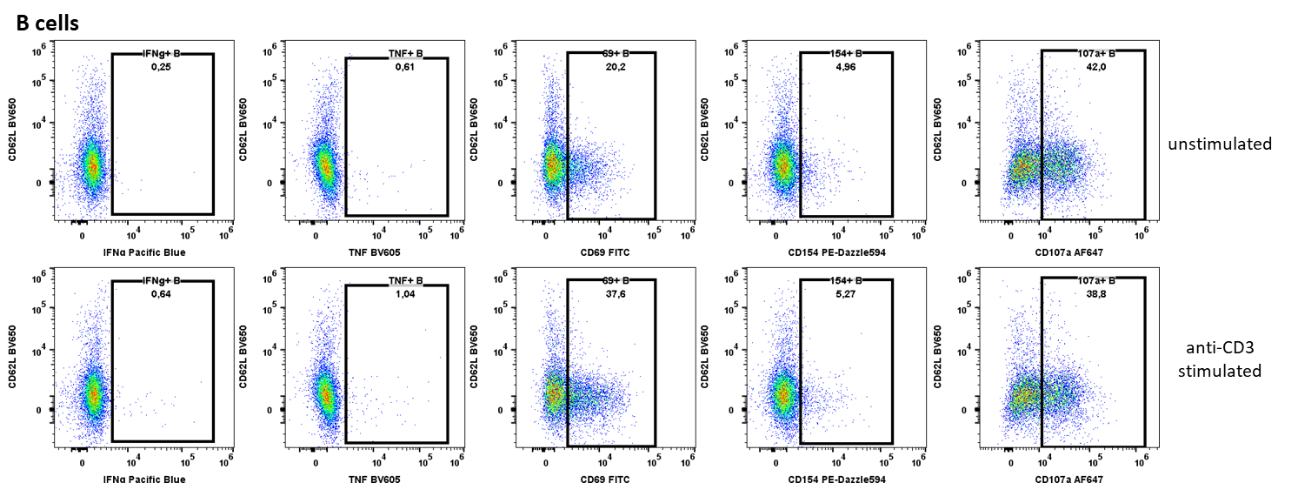


**Figure S1**: **Assessment of activation marker expression on different cell subpopulations (TLAT)**. Manual gating of unstimulated and aCD3 stimulated PBMCs. Expression of activation markers on (A) CD4+ cells, (B) CD8+ cells, (C) Th cells subsets (D) Tfh cells subsets (E) CD4+ maturation subsets (F) CD8+ maturation subsets (G) Other lymphocytes (gdT, MAIT, NKT, NK, B cells). Gating presented in Figure 1.


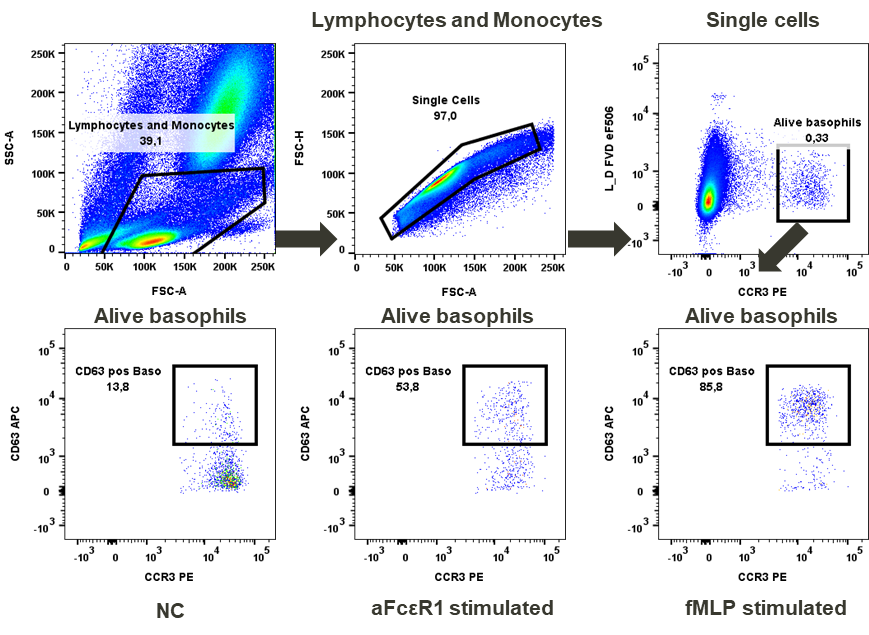


**Figure S2**: **Basophil activation test.** Representative example of a donor with high response. Main gating strategy: Lymphocytes/Monocytes gate is defined in FSC-A vs SSC-A, doublets are excluded in FSC-A vs FSC-H plot. Alive basophils are identified as FVD eF506 negative and CCR3 positive cells. Basophils activation upon stimulation with fMLP or anti-FcεR1 antibodies. Percent of CD63 positive events shows the activation of basophils. NC- negative control.


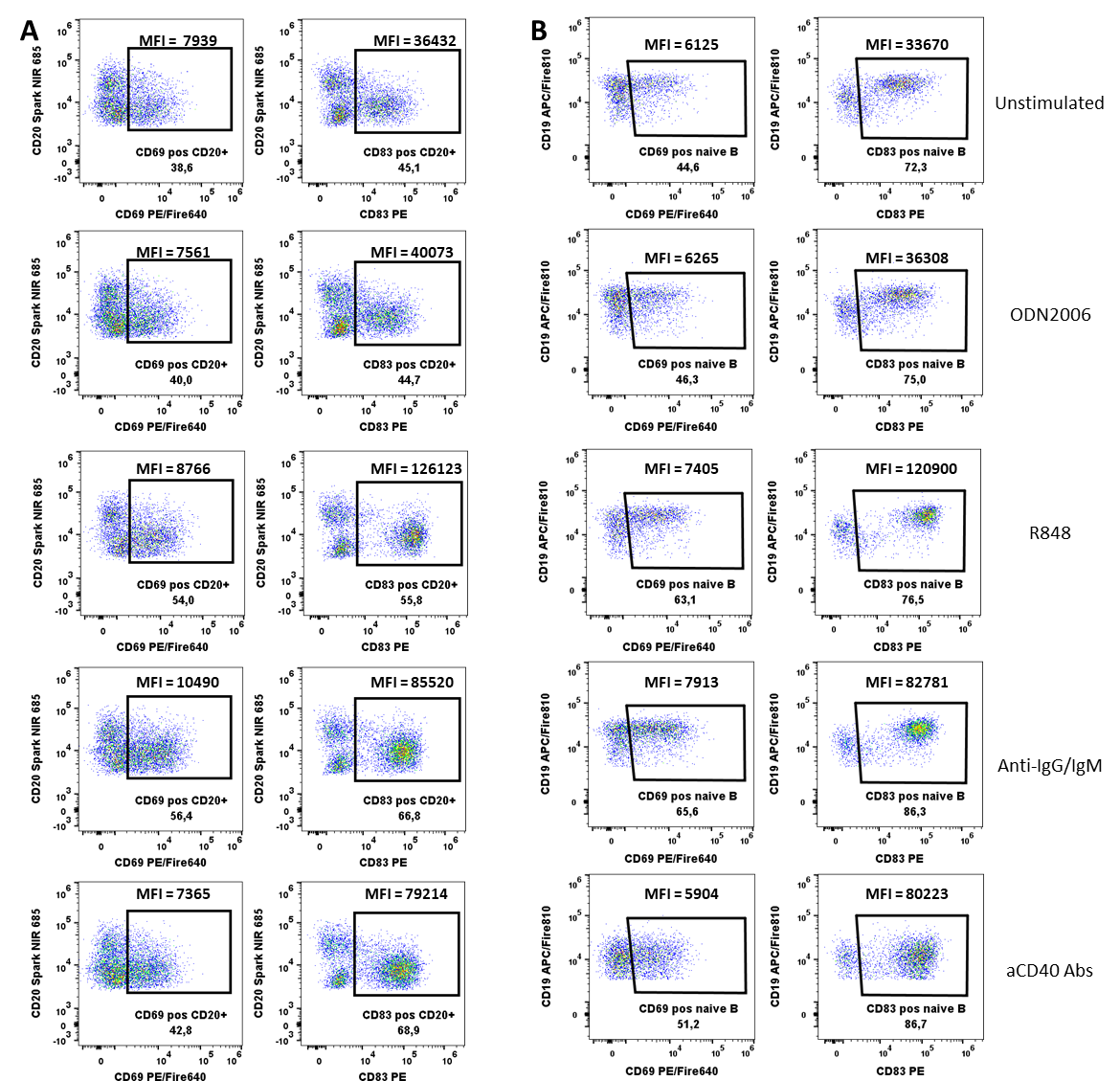


**Figure S3:** **B cells activation test (BLAT).** Expression of activation markers on (**A)** (CD20+ cells) or (**B)** (naïve B cells) cells upon stimulation with anti-IgG/IgM (0.25 µg/ml), R848 (0.5 µg/ml), anti-CD40 Abs (0.25 µg/ml), ODN2006 (0.1 µM). MFI values are provided to estimate the fold induction of a particular activation marker. Gating presented in Figure 6.

**
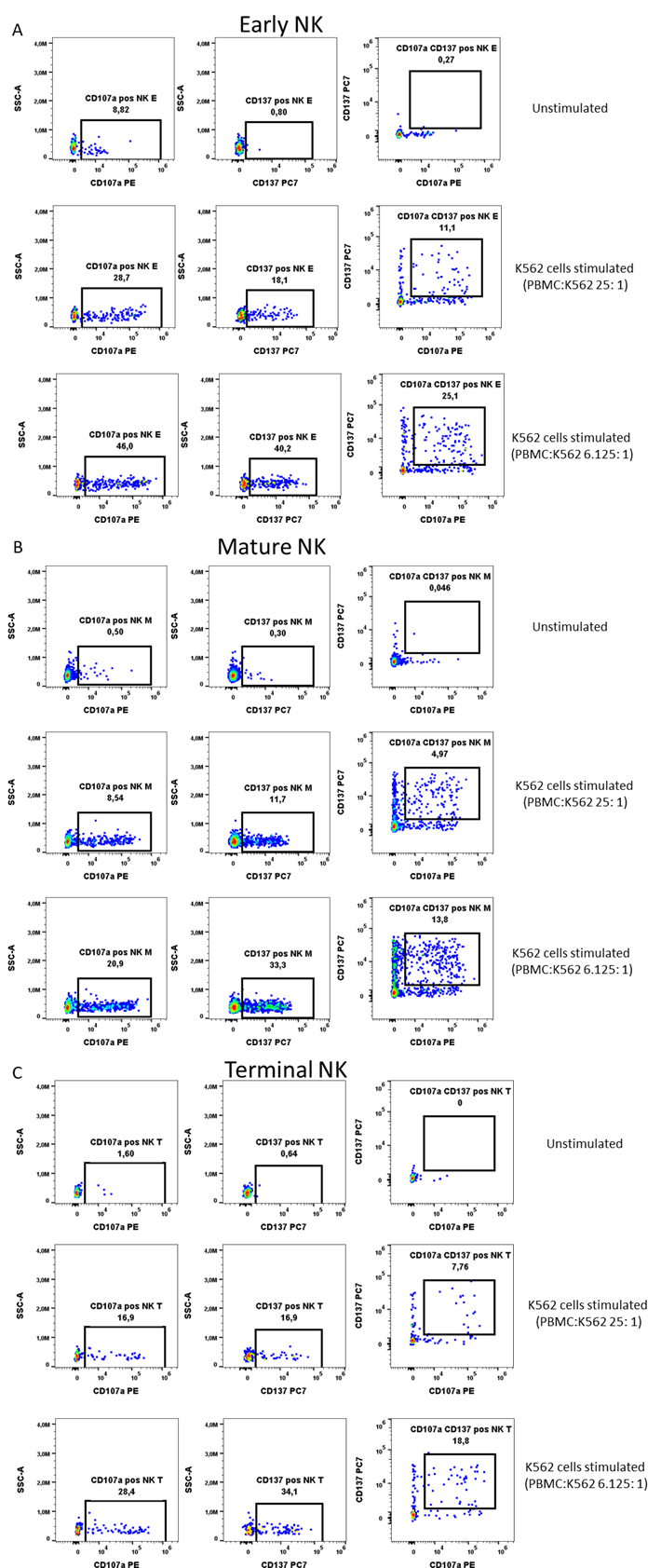
**

**Figure S4: NK cells activation test (NKAT).** Expression of activation markers on (**A)** (early), (**B)** (mature) or (**C)** (terminal) NK cells upon stimulation with K562 cells at ratio 25:1 and 6.25:1 (PBMC:K562). Gating presented in Figure 7.


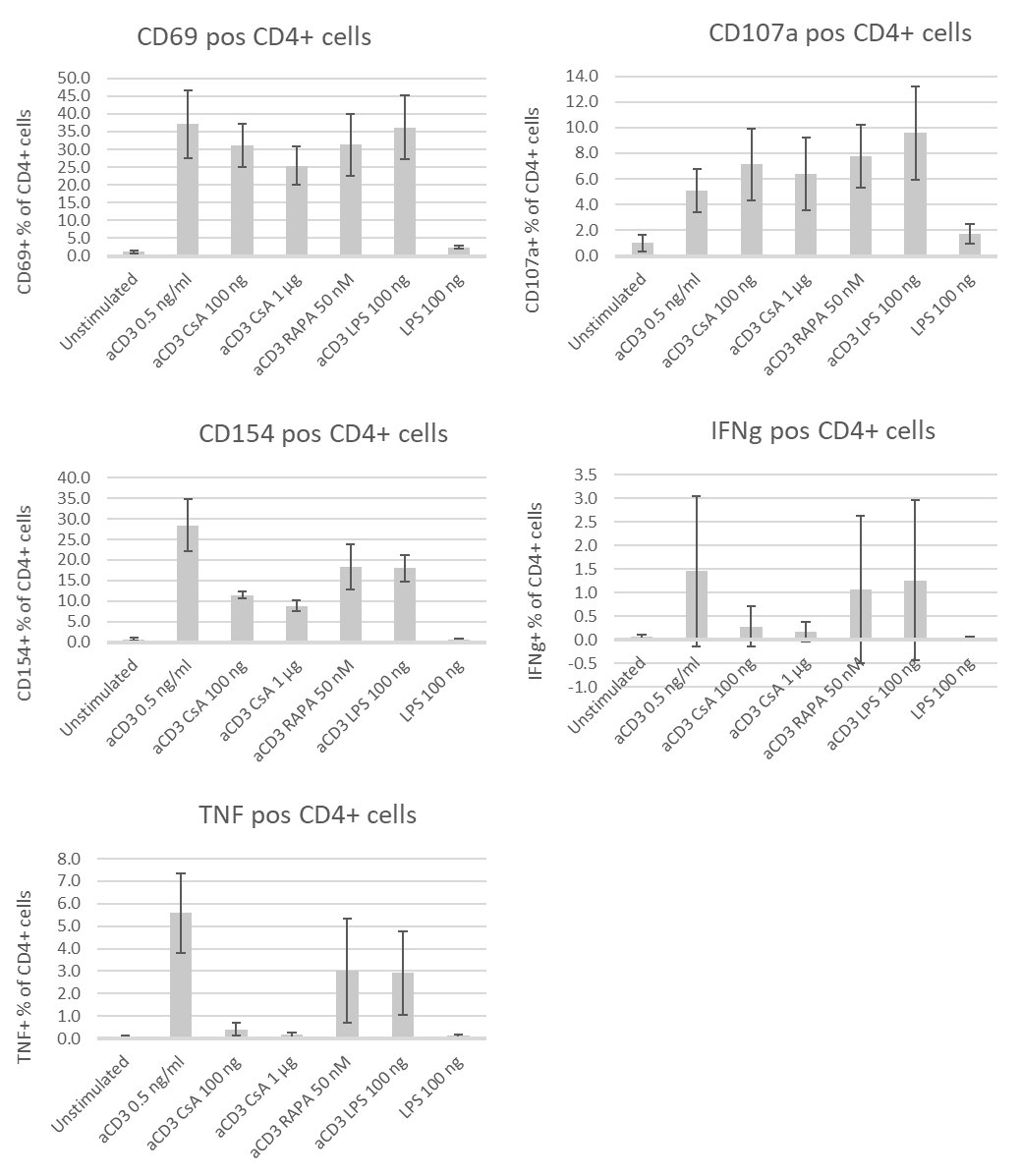


**Figure S5:** Modulation of immune responses with cyclosporin A (CsA), rapamycin (RAPA) and lipopolysaccharide (LPS) in given concentrations in the TLAT assay (stimulation with aCD3). Data of CD4+ T cells are presented, n=4 donors. The percentage of activated cell is shown on the y-axis.


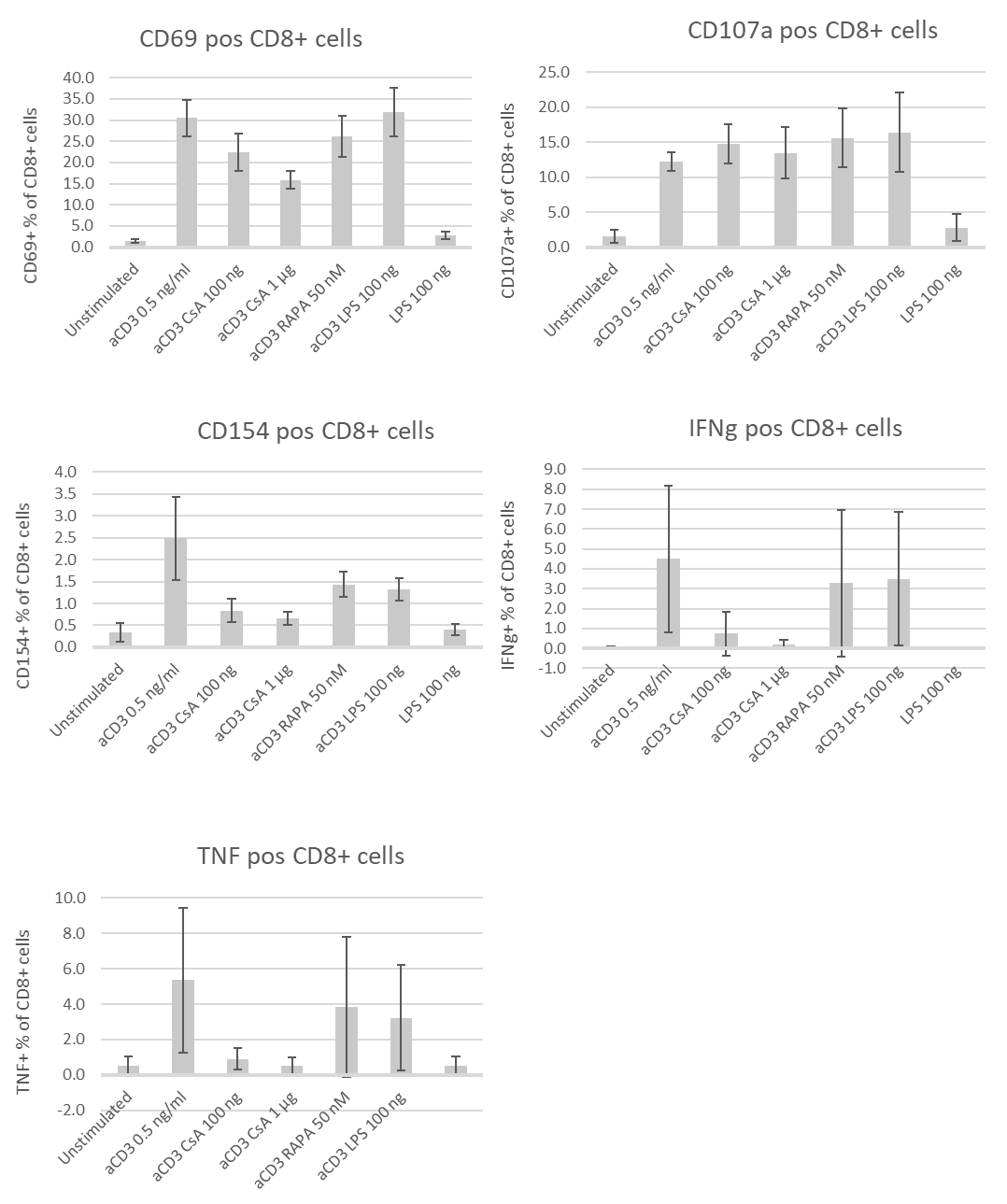


**Figure S6:** Modulation of immune responses with cyclosporin A (CsA), rapamycin (RAPA) and lipopolysaccharide (LPS) in given concentrations in the TLAT assay (stimulation with aCD3). Data of CD8+ T cells are presented, n=4 donors. The percentage of activated cell is shown on the y-axis.


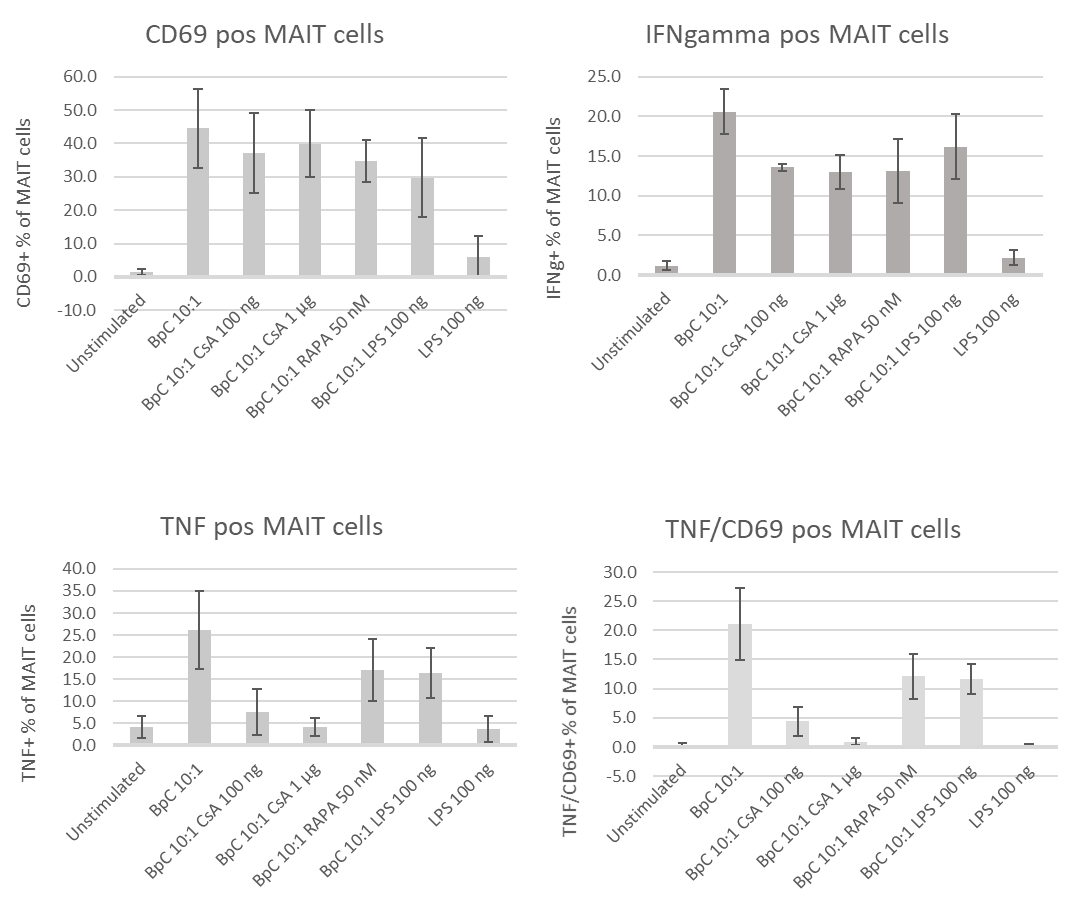


**Figure S7**: Modulation of immune responses with cyclosporin A (CsA), rapamycin (RAPA) and lipopolysaccharide (LPS) in given concentrations in the MAT assay (stimulation with 10:1 BpC). Data of MAIT cells are presented, n=4 donors. The percentage of activated cell is shown on the y-axis.


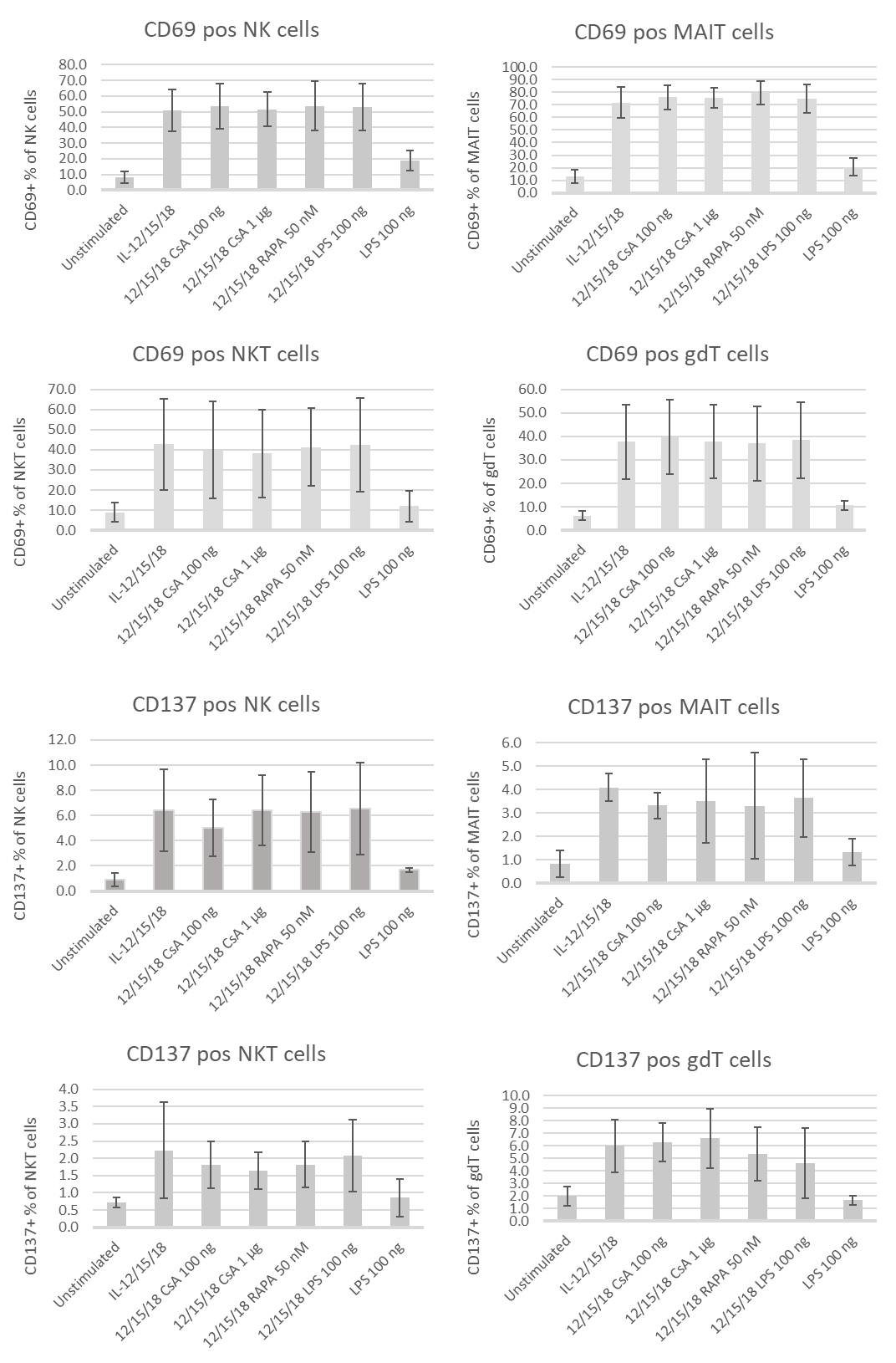


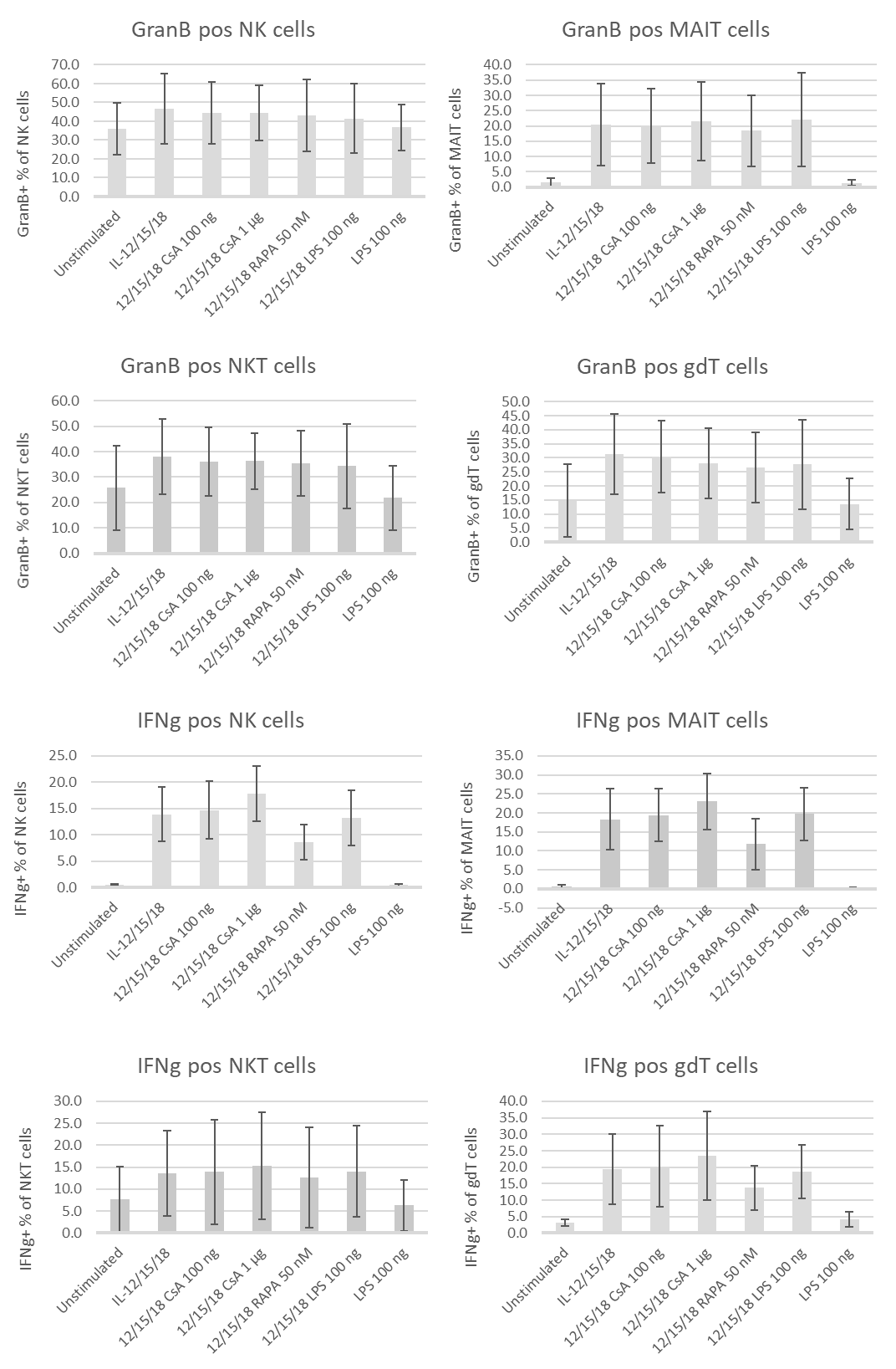


**Figure S8**: Modulation of immune responses with cyclosporin A (CsA), rapamycin (RAPA) and lipopolysaccharide (LPS) in given concentrations in the CAT assay (stimulation with IL12/15/18), n=4 donors. The names of the cell populations are shown in the graph headings. The percentage of activated cell is shown on the y-axis.

**
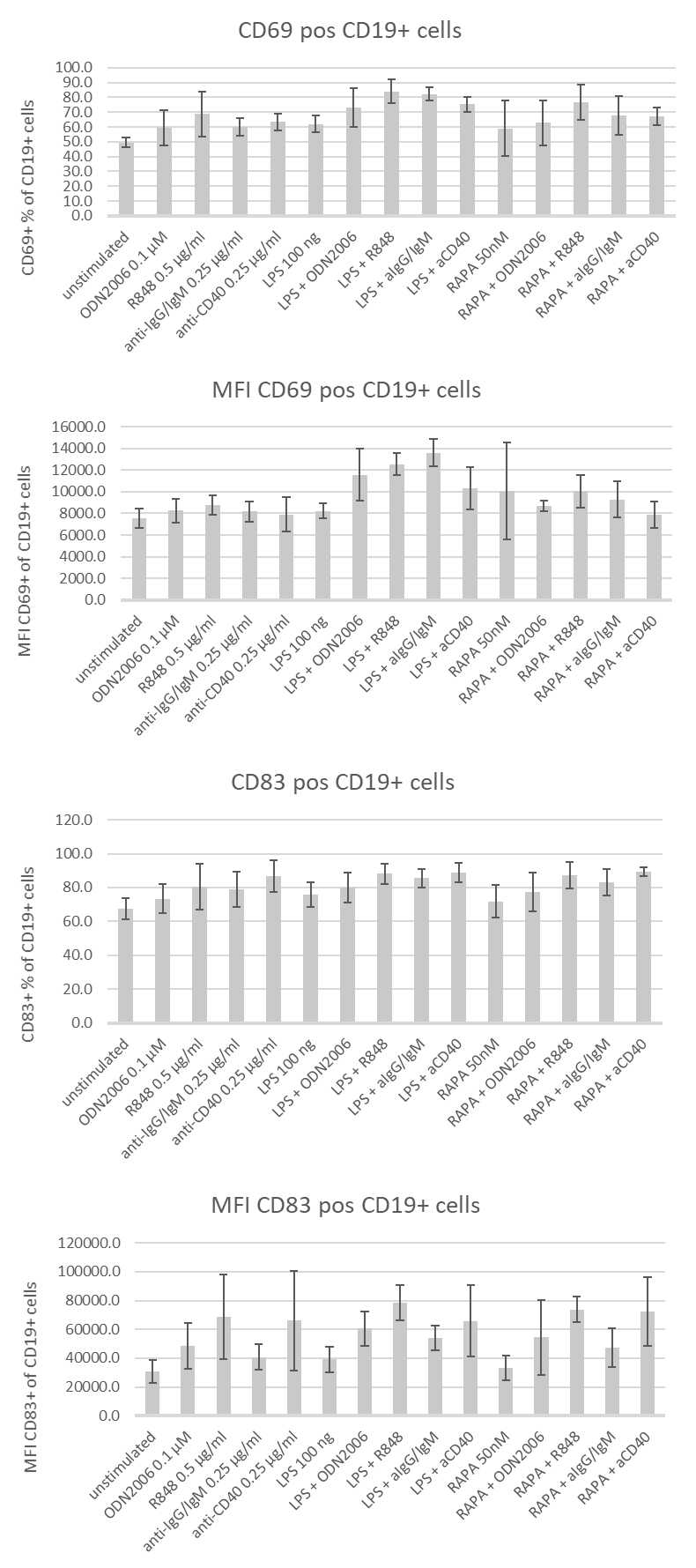
Figure S9**: Modulation of immune responses with cyclosporin A (CsA), rapamycin (RAPA) and lipopolysaccharide (LPS) in given concentrations in the BLAT assay (stimulation with CpG ODN2006, R848, anti-IgG/IgM, anti-CD40), n=4 donors. The names of the cell populations are shown in the graph headings. The percentage of activated cells or shift in MFI is shown on the y-axis.

**
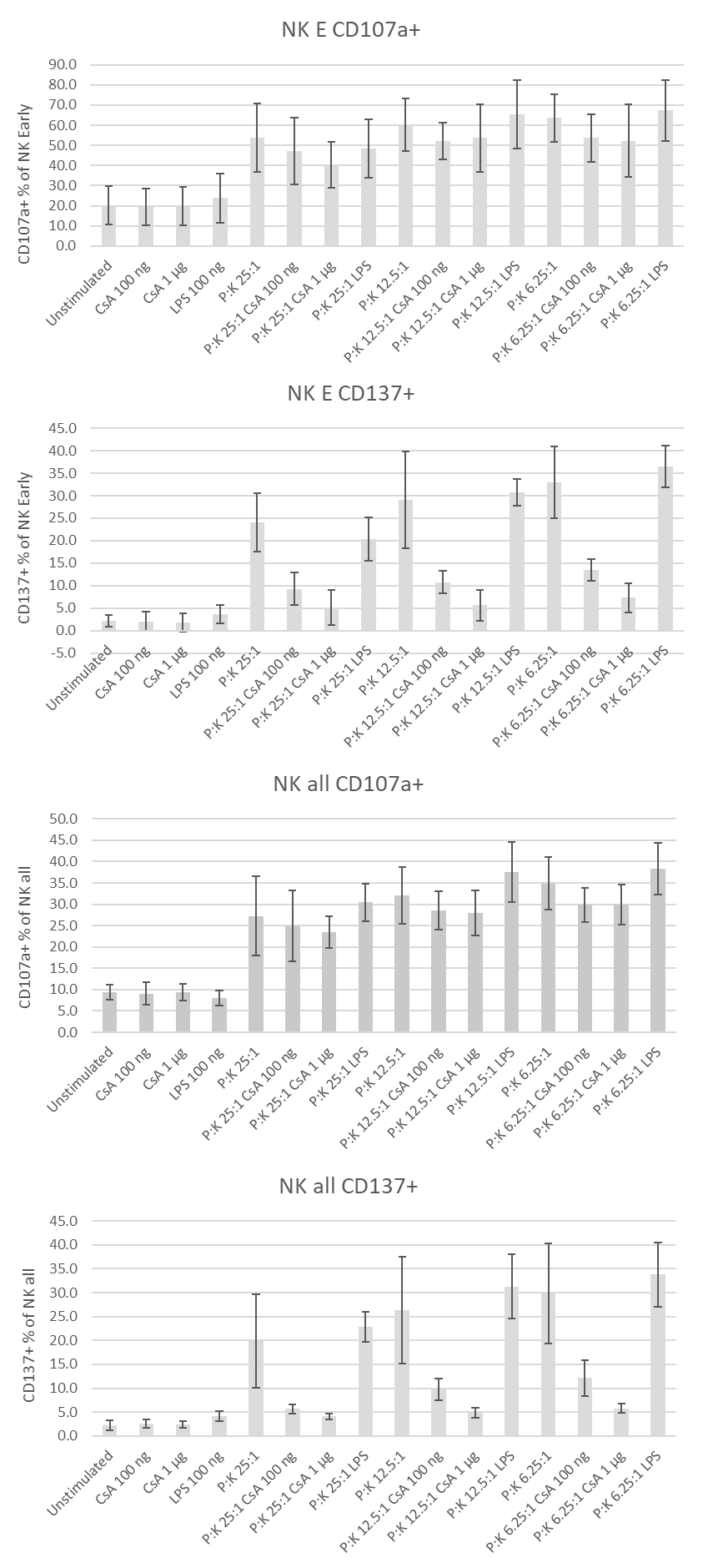
Figure S10**: Modulation of immune responses with cyclosporin A (CsA), rapamycin (RAPA) and lipopolysaccharide (LPS) in given concentrations in the NKAT assay (stimulation with PBMC:K562 ratio 25:1 and 6.25:1), n=4 donors. The names of the cell populations are shown in the graph headings. The percentage of activated cell is shown on the y-axis. Definition of NK cells as in Figure 7, E=early. Similar results were obtained for mature and terminal NK cells, data not shown.


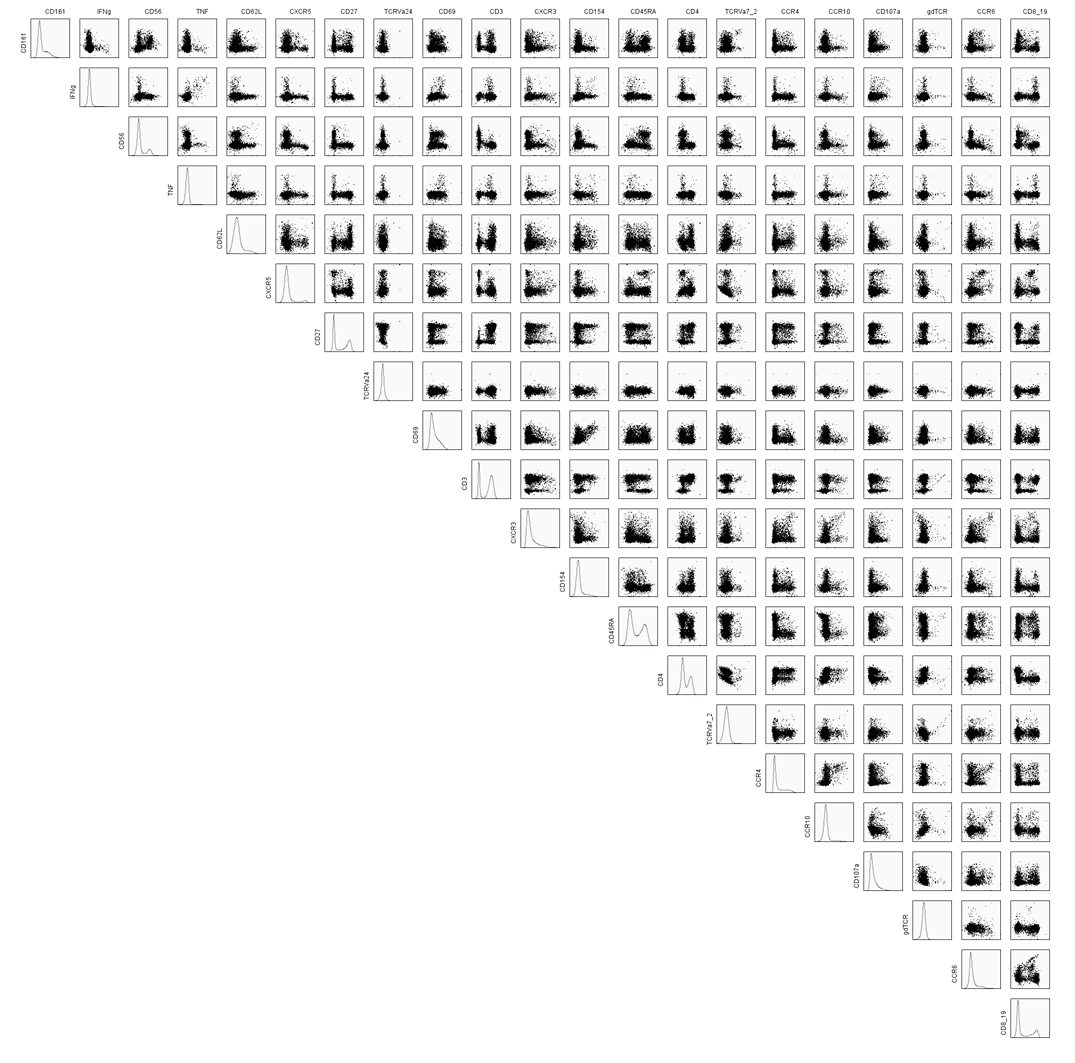
**Figure S11: N-by-N bivariate plots of all markers combinations in TLAT.** Cells were gated on lymphocytes. All markers listed in Table S2 except for Zombie NIR (viability marker excluded from further data analysis) are presented.


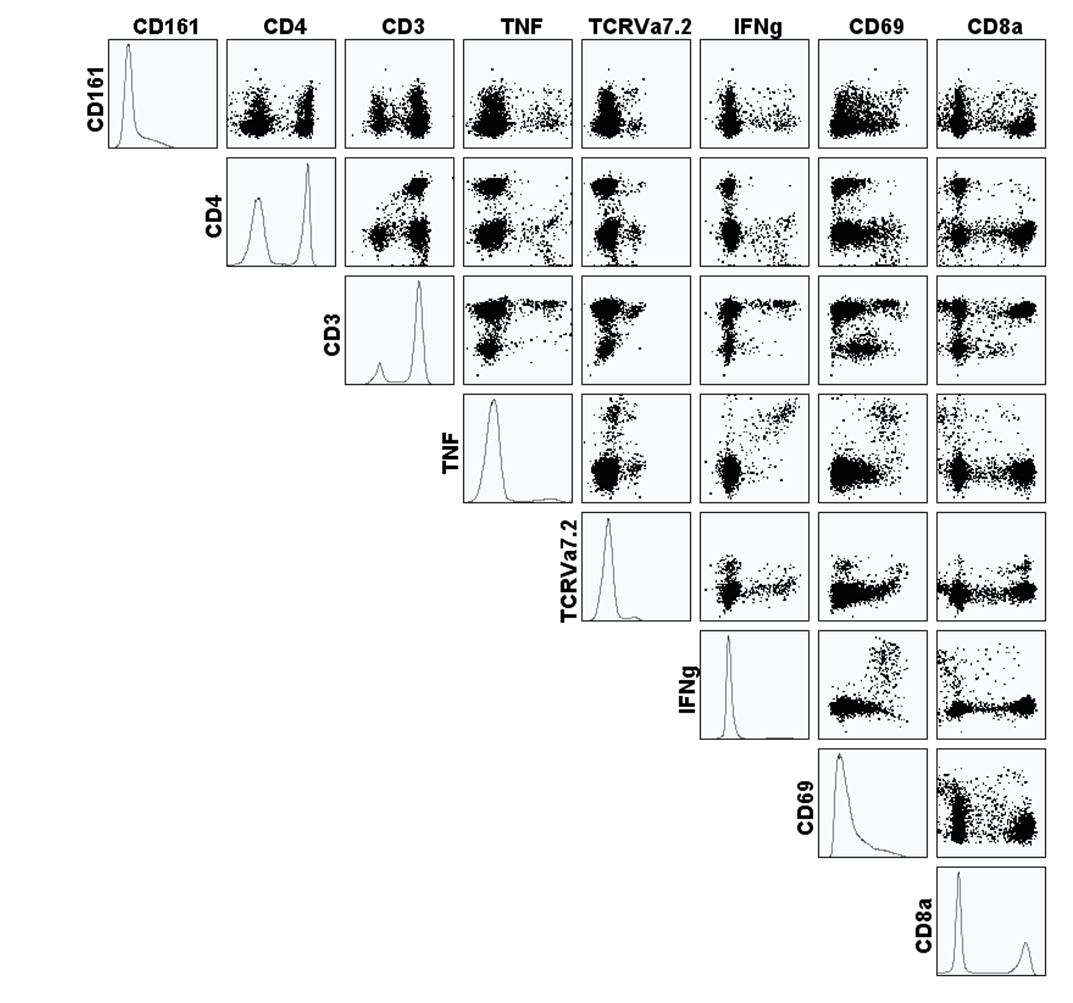
**Figure S12: N-by-N bivariate plots of all markers combinations in MAT.** Cells were gated on lymphocytes. All markers listed in Table S3 except for Zombie NIR (viability marker excluded from further data analysis) are presented.

**
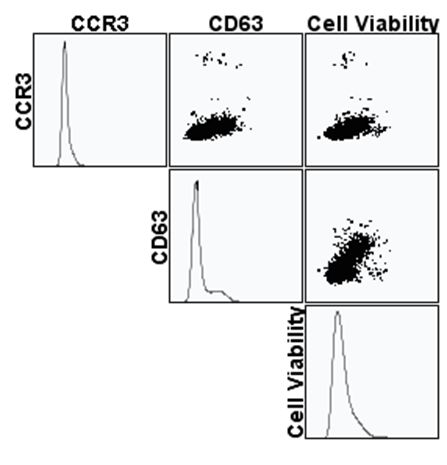
**

**Figure S13: N-by-N bivariate plots of all markers combinations in BAT.** Cells were gated on lymphocytes and monocytes. All markers listed in Table S4 are presented.

**Figure S14: N-by-N bivariate plots of all markers combinations in CAT.** Cells were gated on lymphocytes. All markers listed in Table S5 except for Zombie NIR (viability marker excluded from further data analysis) are presented.
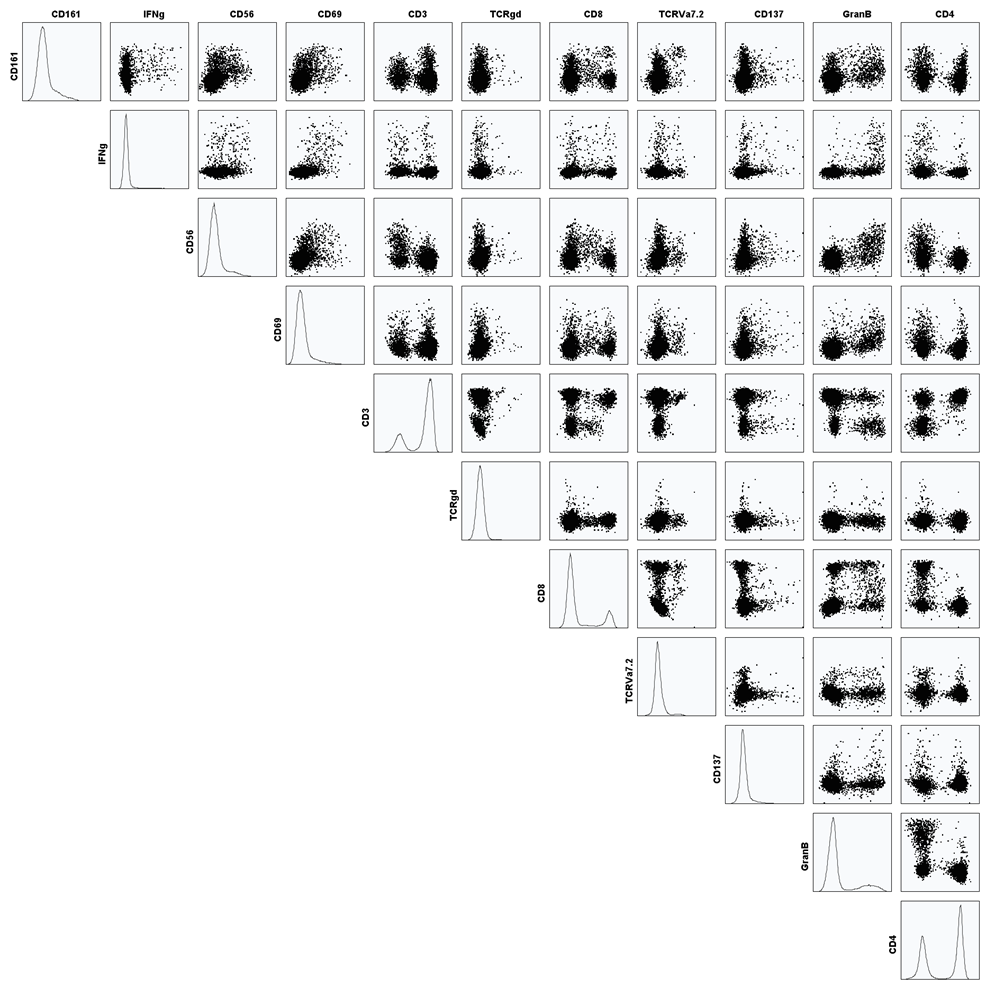


**Figure S15: N-by-N bivariate plots of all markers combinations in BLAT.** Cells were gated on lymphocytes. All markers listed in Table S6 except for Zombie NIR (viability marker excluded from further data analysis) are presented.
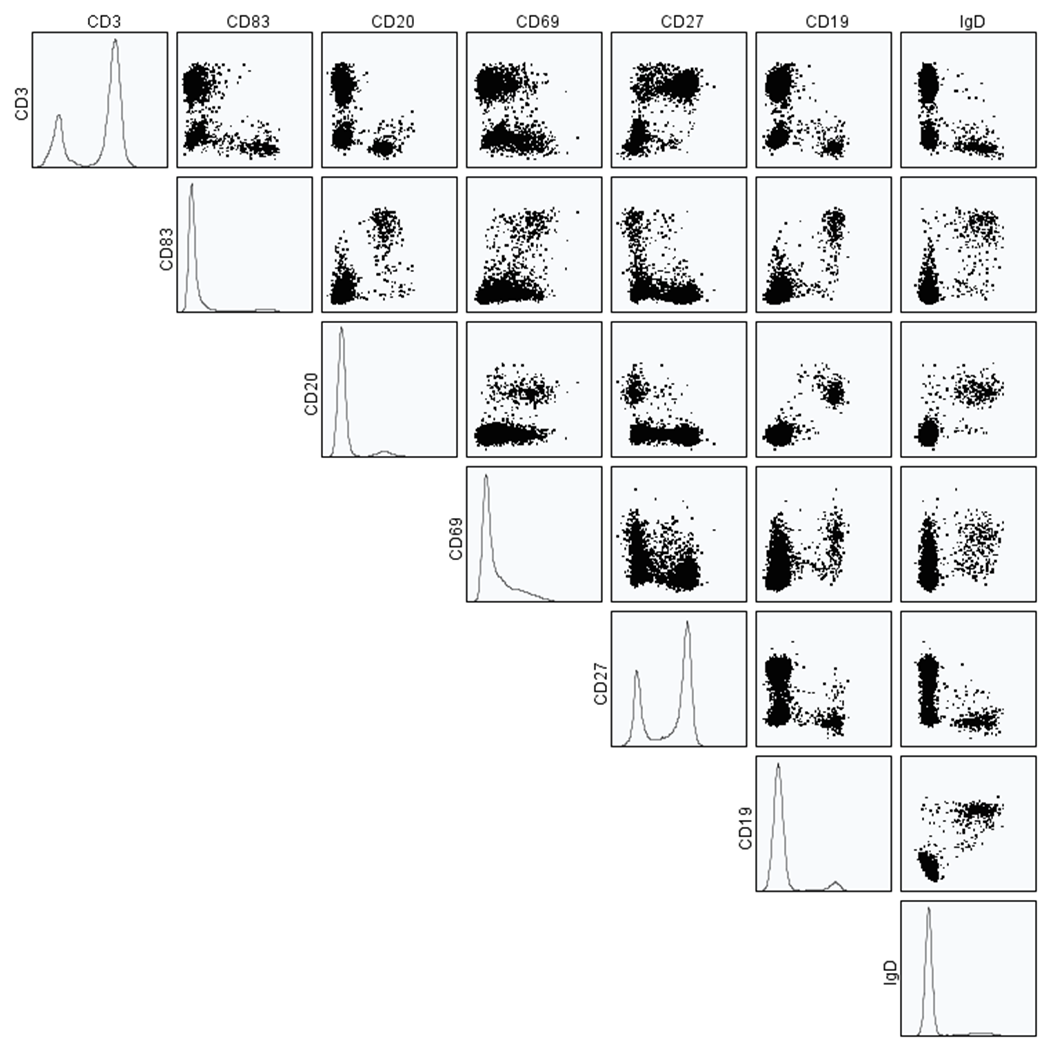


**Figure S16: N-by-N bivariate plots of all markers combinations in NKAT.** Cells were gated on lymphocytes. All markers listed in Table S7 except for Zombie NIR (viability marker excluded from further data analysis) are presented
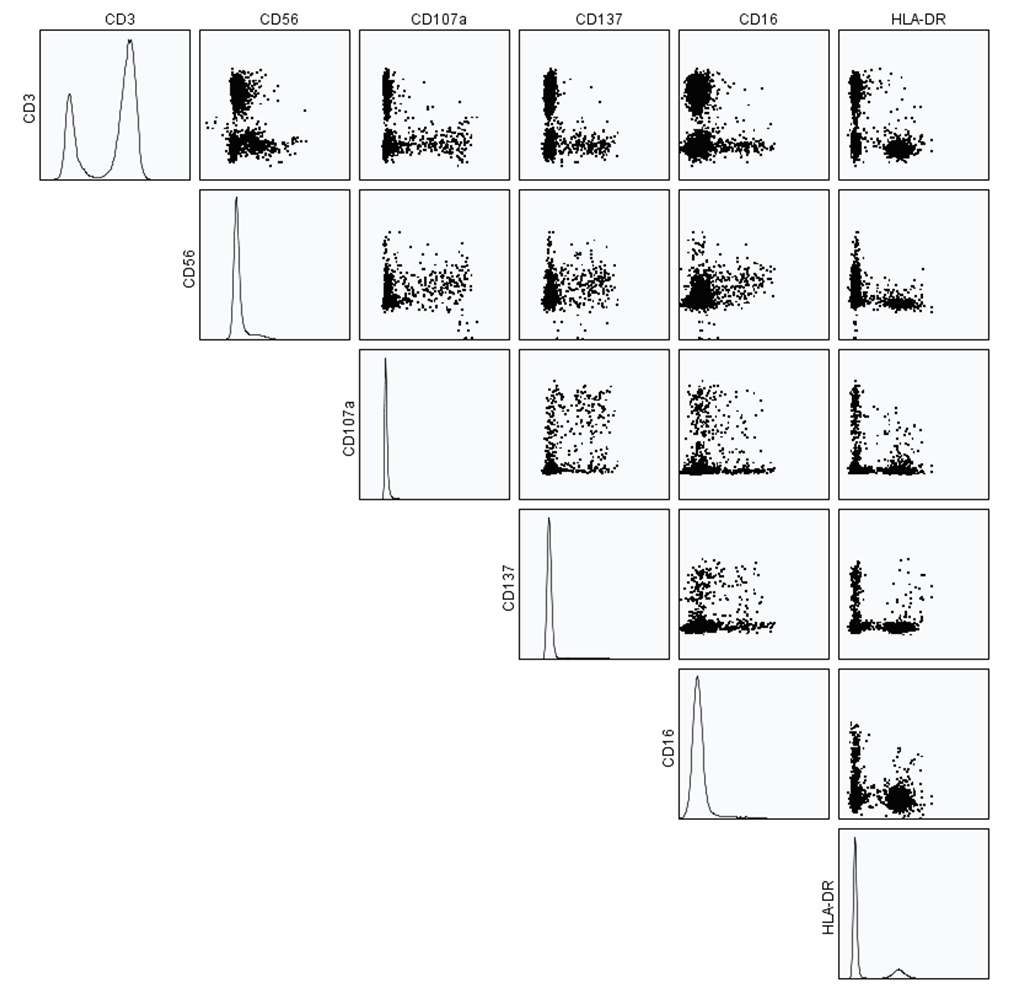
.

**Spectral unmixing**

The unmixing accuracy is highly dependent on the quality of the reference controls and their ability to accurately represent the spectra of fluorochromes present in the Multicolor staining. Using a full spectrum flow cytometer allows detection of even the smallest differences in fluorochrome emission. As there may be a difference in spectrum of the fluorochrome bound to beads and to the cells, we attempted to use the cells for most fluorochromes and use the beads only in the case when we could not collect enough events for positively stained cells. In general, the selected cells and beads combination gave a satisfactory fluorochrome spectral unmixing result which is shown in N-by-N bivariate plots for each antibody panel – Figure S11-S16. Moreover N-by-N plots show that the selected fluorochrome combination for each panel does not contribute to increased spreading errors which could limit positive population detection of markers being combined with fluorochromes being the most affected by spreading error from other fluorochromes. Especially for TLAT antibody panel it was design in the way to limit any influence of spreading error on proper positive marker identification.

**Supplementary Tables**

**Table S1:** Detailed summary of the in vitro immune cell activation methods showing stimulus used, target cells (PBMC), proposed preincubation time with chemicals, in vitro stimulation time, number of markers included in the flow cytometry panel and a list of activation markers for estimation of effect of chemicals on immune response.

| **Activation test acronym** | **Stimulus** | **Target cells** | **Biological material** | **Preincubation with chemical (h)** | **Stimulation time (h)** | **Readout panel**  **No. of markers** | **Activation markers** |
| --- | --- | --- | --- | --- | --- | --- | --- |
| TLAT | aCD3 Abs | Th, Tc, NKT, MAIT, gdT (including Th subtypes) | PBMC | 24 | 6 | 23 | CD69, CD107a, CD154, TNF, IFNg |
| MAT | E.coli | MAIT, Tc, NKT, gdT | PBMC | 24 | 6 | 9 | TNF, IFNg, CD69 |
| BAT | fMLP  FcεR1 Abs/IL-3 | Basophils | Whole blood | 1 | 0.5 | 3 | CD63 |
| CAT | IL-12/15/18 | MAIT, NK, NKT, gdT | PBMC | 24 | 20 | 12 | IFNg, CD69, CD137, GranB |
| BLAT | ODN2006  R848  aCD40 Abs  Anti-IgM/IgG | B cells | PBMC | 24 | 4 | 8 | CD69, CD83 |
| NKAT | K562 cells | NK cells | PBMC | 24 | 6 | 7 | CD137, CD107a |

**Table S2**: Antibodies used to stain lymphocytes in the TLAT assay for flow cytometry analysis.

| Specificity | Fluorophore | Clone | Catalog# | Vendor | Titer per 10^6^ cells | Titer  (ng/100 µl) | Staining condition |
| --- | --- | --- | --- | --- | --- | --- | --- |
| CD3 | SparkBlue^TM^ 550 | SK7 | 344852 | BioLegend | 1:200 | 100 | Extracellular |
| CD4 | PerCP | SK3 | 344624 | BioLegend | 1:100 | 25 | Extracellular |
| CD8 | APC/Fire^TM^ 810 | SK1 | 344764 | BioLegend | 1:800 | 6.25 | Extracellular |
| CD19 | APC/Fire^TM^ 810 | HIB19 | 302272 | BioLegend | 1:400 | 12.5 | Extracellular |
| CD27 | Brilliant Violet 750^TM^ | O323 | 302850 | BioLegend | 1:200 | 50 | Extracellular |
| CD45RA | PE/Fire640 | HI100 | 301169 | BioLegend | 1:200 | 3 | Extracellular |
| CD56 | Brilliant Violet 510^TM^ | HCD56 | 318340 | BioLegend | 1:200 | 50 | Extracellular |
| CD62L | Brilliant Violet 650^TM^ | DREG-56 | 304832 | BioLegend | 1:400 | 20 | Extracellular |
| CD161 | Brilliant Violet 421^TM^ | HP-3G10 | 339914 | BioLegend | 1:100 | 100 | Extracellular |
| CCR4 | PE-Cyanine7 | I.291H4 | 359410 | BioLegend | 1:400 | 50 | Extracellular |
| CCR6 | APC-Cyanine7 | G034E3 | 353432 | BioLegend | 1:200 | 50 | Extracellular |
| CCR10 | APC | REA326 | 130-120-406 | Miltenyi | 1:200 | - | Extracellular |
| CXCR3 | PE | G025H7 | 353706 | BioLegend | 1:200 | 50 | Extracellular |
| CXCR5 | Brilliant Violet 711^TM^ | J252D4 | 356934 | BioLegend | 1:200 | 50 | Extracellular |
| TCRVα7.2 | PerCP-Cyanine5.5 | 3C10 | 351710 | BioLegend | 1:100 | 200 | Extracellular |
| TCRVα24 | Brilliant Violet 785^TM^ | 6B11 | 342932 | BioLegend | 1:200 | 25 | Extracellular |
| TCRγδ | Alexa Fluor 660 | B1 | 331239 | BioLegend | 1:100 | 200 | Extracellular |
| CD69 | FITC | FN50 | 310904 | BioLegend | 1:200 | 50 | Intracellular |
| CD107a | Alexa Fluor^®^ 647 | H4A3 | 328612 | BioLegend | 1:1600 | 12.5 | In medium |
| CD154 | PE-Dazzle594 | 24-31 | 310840 | BioLegend | 1:400 | 37.5 | Intracellular |
| IFNγ | Pacific Blue | 4S.B3 | 502522 | BioLegend | 1:400 | 125 | Intracellular |
| TNF | Brilliant Violet 605^TM^ | MAb11 | 502936 | BioLegend | 1:400 | 25 | Intracellular |
| Fixable viability dye | Zombie NIR^TM^ |  | 423106 | BioLegend | 1:3000 | - | Extracellular |

**Table S3:** Antibodies used to stain lymphocytes in MAT for flow cytometry analysis.

| Specificity | Fluorophore | Clone | Catalog# | Vendor | Titer per 10^6^ cells | Titer  (ng/100 µl) | Staining condition |
| --- | --- | --- | --- | --- | --- | --- | --- |
| CD3 | FITC | UCHT-1 | 300406 | BioLegend | 1:200 | 100 | Extracellular |
| CD4 | BV510 | SK3 | 344634 | BioLegend | 1:200 | 12.5 | Extracellular |
| CD8a | APC-eFluor780 | RPA-T8 | 47-0088-42 | ThermoFisher | 1:400 | 6.25 | Extracellular |
| CD161 | Brilliant Violet 421^TM^ | HP-3G10 | 339914 | BioLegend | 1:200 | 50 | Extracellular |
| TCRVα7.2 | PerCP-Cyanine5.5 | 3C10 | 351710 | BioLegend | 1:200 | 100 | Extracellular |
| CD69 | APC | FN50 | 310910 | BioLegend | 1:400 | 25 | Intracellular |
| IFNγ | PE-Cyanine7 | B27 | 506518 | BioLegend | 1:800 | 12.5 | Intracellular |
| TNF | PE | MAb11 | 502909 | BioLegend | 1:800 | 12.5 | Intracellular |
| Fixable viability dye | Zombie NIR^TM^ |  | 423106 | BioLegend | 1:3000 | - | Extracellular |

| Specificity | Fluorophore | Clone | Catalog# | Vendor | Titer per 10^6^ cells | Titer  (ng/100 µl) | Staining condition |
| --- | --- | --- | --- | --- | --- | --- | --- |
| CCR3 | PE | 5E8 | 310706 | BioLegend | 1:200 | 50 | Extracellular |
| CD63 | APC | H5C6 | 353008 | BioLegend | 1:400 | 50 | Extracellular |
| Fixable viability dye | eFluor^TM^ 506 |  | 65-0866-18 | eBiosciences | 1:750 | - | Extracellular |

**Table S4**: Antibodies used to stain lymphocytes in BAT for flow cytometry analysis.

**Table S5**: Antibodies used to stain lymphocytes in CAT for flow cytometry analysis.

| Specificity | Fluorophore | Clone | Catalog# | Vendor | Titer per 10^6^ cells | Titer  (ng/100 µl) | Staining condition |
| --- | --- | --- | --- | --- | --- | --- | --- |
| CD3 | SparkBlue^TM^ 550 | SK7 | 344852 | BioLegend | 1:200 | 100 | Extracellular |
| CD4 | APC-eFluor780 | RPA-T4 | 47-0049-42 | ThermoFisher | 1:200 | 50 | Extracellular |
| CD8 | PerCP | SK1 | 344708 | BioLegend | 1:200 | 100 | Extracellular |
| CD56 | Brilliant Violet 510^TM^ | HCD56 | 318340 | BioLegend | 1:200 | 50 | Extracellular |
| CD161 | Brilliant Violet 421^TM^ | HP-3G10 | 339914 | BioLegend | 1:200 | 50 | Extracellular |
| TCRVα7.2 | PerCP-Cyanine5.5 | 3C10 | 351710 | BioLegend | 1:200 | 100 | Extracellular |
| TCRγδ | PE | B1 | 331210 | BioLegend | 1:200 | 200 | Extracellular |
| CD69 | FITC | FN50 | 310904 | BioLegend | 1:200 | 50 | Intracellular |
| CD137 | APC | 4B4-1 | 309810 | BioLegend | 1:200 | 50 | Intracellular |
| IFNγ | Pacific Blue | 4S.B3 | 502522 | BioLegend | 1:400 | 125 | Intracellular |
| GranzymeB | Alexa Fluor® 700 | QA16A02 | 372222 | BioLegend | 1:200 | 12.5 | Intracellular |
| Fixable viability dye | Zombie NIR^TM^ |  | 423106 | BioLegend | 1:3000 | - | Extracellular |

**Table S6:** Antibodies used to stain lymphocytes in BLAT for flow cytometry analysis.

| Specificity | Fluorophore | Clone | Catolog# | Vendor | Titer per 10^6^ cells | Titer  (ng/100 µl) | Staining  condition |
| --- | --- | --- | --- | --- | --- | --- | --- |
| CD3 | Pacific Blue | UCHT-1 | 300431 | BioLegend | 1:100 | 100 | Extracellular |
| CD19 | APC/Fire^TM^ 810 | HIB19 | 302272 | ThermoFisher | 1:400 | 12.5 | Extracellular |
| CD20 | Spark NIR™ 685 | 2H7 | 302365 | BioLegend | 1:800 | 12.5 | Extracellular |
| CD27 | Brilliant Violet 750^TM^ | O323 | 302850 | BioLegend | 1:400 | 25 | Extracellular |
| CD69 | PE/Fire^TM^ 640 | FN50 | 310960 | BioLegend | 1:400 | 12.5 | Extracellular |
| CD83 | PE | HB15e | 305307 | BioLegend | 1:400 | 75 | Extracellular |
| IgD | APC/Fire^TM^ 750 | IA6-2 | 348237 | BioLegend | 1:400 | 12.5 | Extracellular |
| Fixable viability dye | Zombie NIR^TM^ |  | 423106 | BioLegend | 1:3000 | - | Extracellular |

**Table S7**: Antibodies used to stain lymphocytes in NKAT for flow cytometry analysis.

| Specificity | Fluorophore | Clone | Catalog# | Vendor | Titer per 10^6^ cells | Titer  (ng/100 µl) | Staining condition |
| --- | --- | --- | --- | --- | --- | --- | --- |
| CD3 | Pacific Blue | UCHT-1 | 300431 | BioLegend | 1:100 | 100 | Extracellular |
| CD16 | Alexa Fluor® 700 | 3G8 | 302026 | BioLegend | 1:400 | 25 | Extracellular |
| CD56 | Brilliant Violet 510^TM^ | HCD56 | 318340 | BioLegend | 1:200 | 50 | Extracellular |
| HLA-DR | APC/Fire^TM^ 810 | L243 | 307674 | BioLegend | 1:400 | 12.5 | Extracellular |
| CD107a | PE | H4A3 | 328608 | BioLegend | 1:1600 | 12.5 | In medium |
| CD137 | PE-Cyanine7 | 4B4-1 | 309818 | BioLegend | 1:400 | 25 | Intracellular |
| Fixable viability dye | Zombie NIR^TM^ |  | 423106 | BioLegend | 1:3000 | - | Extracellular |

**
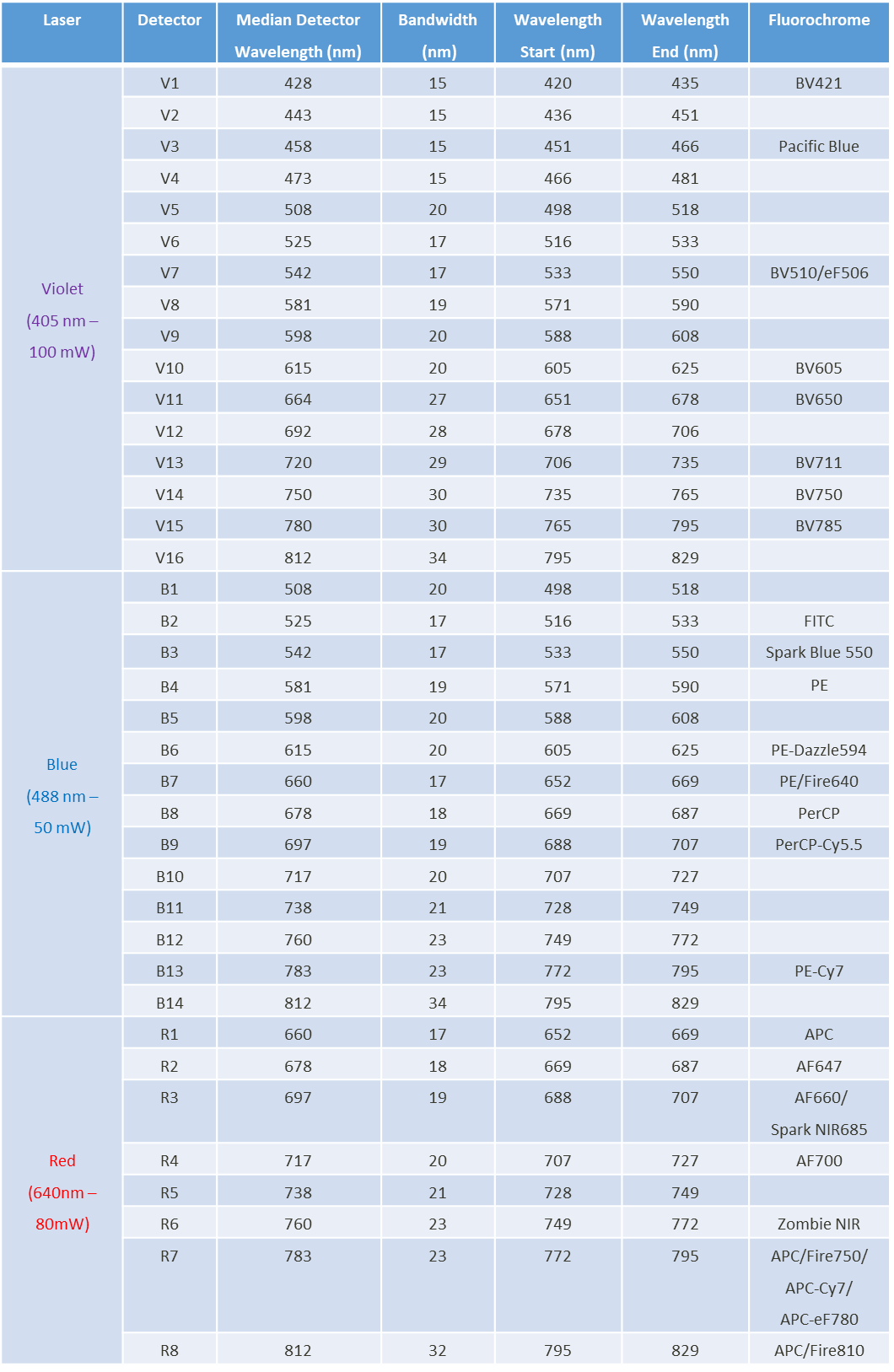
Table S8:** Fluorochrome – detector assignment for 3L Cytek Aurora for all fluorochromes used in all antibody panels. The table shows which lasers are necessary for optimal fluorochrome excitation. In case of TLAT, where 22 different fluorochromes are applied, full spectral flow cytometer should be used^#^. For all other antibody panels conventional 3-laser flow cytometry instruments can be used as well.

^#^Please note that in the case of the TLAT assay, for the selection of the 22 different fluorochromes, we have chosen some dyes such as Pacific Blue and BV421 or APC and Alexa Fluor 647 and Alexa Fluor 660, which are difficult to distinguish by conventional flow cytometry where the fluorochromes are detected in their peak emission channel. Spectral flow cytometry makes it much easier to differentiate between fluorochromes, as the entire spectrum of fluorochromes is considered when detecting different fluorochromes, allowing even very similar fluorochromes to be clearly identified.

**Table S9:** Individual results and mean ± SD of the data (% of activated cells or MFI) gained in the TLAT, MAT, BAT, CAT, BLAT and NKAT assays after stimulation with the corresponding stimulus. Calculation of sample size^#^ for an expected mean reduction of 25% and 50%. Sample sizes below 15 are highlighted in grey.

**Table S9: continued**

^#^Calculations have been performed with the formula provided in the link: [Compare 2 Means 2-Sample, 2-Sided Equality | Power and Sample Size Calculators | HyLown](http://powerandsamplesize.com/Calculators/Compare-2-Means/2-Sample-Equality), referenced in Chow S, Shao J, Wang H. 2008. Sample Size Calculations in Clinical Research. 2nd Ed. Chapman & Hall/CRC Biostatistics Series. **page 58**.
